# Dielectric polarization and charge transport in Gd-doped Fe_3_O_4_ nanocrystals for energy storage and electronic devices

**DOI:** 10.1039/d6ra06350k

**Published:** 2026-09-28

**Authors:** Neeraj Kadian, Mutaz Salih, Renu Kumari, Smita Korpal, Jasvir Dalal, Ahmed B. M. Ibrahim

**Affiliations:** a Department of Physics, Government College for Women Rohtak-124001 India; b Department of Physics, Rajdhani College, University of Delhi Delhi-110015 India jasvirdalal2012@gmail.com; c Department of Chemistry, College of Science, Imam Mohammad Ibn Saud Islamic University (IMSIU) Riyadh 11623 Saudi Arabia; d Department of Chemistry, Faculty of Science, Assiut University Assiut 71516 Egypt

## Abstract

Gd-doped magnetite (Gd_*x*_Fe_3−*x*_O_4_) nanocrystals were synthesized *via* the co-precipitation method with an aim to study the effect of rare-earth substitution on their structure, shape, and dielectric and magnetic properties. Structural analysis using X-ray diffraction revealed that the samples formed a single-phase cubic spinel structure with minor lattice distortion upon Gd substitution. Nanocrystalline materials were characterized as cluster-like grains using field-emission scanning electron microscopy. Fourier transform infrared spectroscopic analysis provided evidence of a spinel structure and revealed metal–oxygen vibrations at the tetrahedral and octahedral sites. From the dielectric analysis, improved dielectric permittivity at lower frequencies, attributed to Maxwell–Wagner interfacial polarization, and lower dielectric loss at higher frequencies indicate their potential for dielectric and energy-related applications. The application of an alternating current to the materials revealed conductivity consistent with the thermally activated hopping mechanism and the correlated barrier hopping model. Magnetic analysis revealed a soft magnetic material with lower coercivity and moderate saturation magnetization. The combined structural, dielectric, electrical, and magnetic characteristics highlight the potential of Gd-doped magnetite nanocrystals for future energy-related and electronic applications.

## Abstract 2

Gd-doped magnetite (Gd_*x*_Fe_3−*x*_O_4_) nanocrystals were synthesized *via* the co-precipitation method with an aim to study the effect of rare-earth substitution on their structure, shape, and dielectric and magnetic properties.

## Graphical abstract

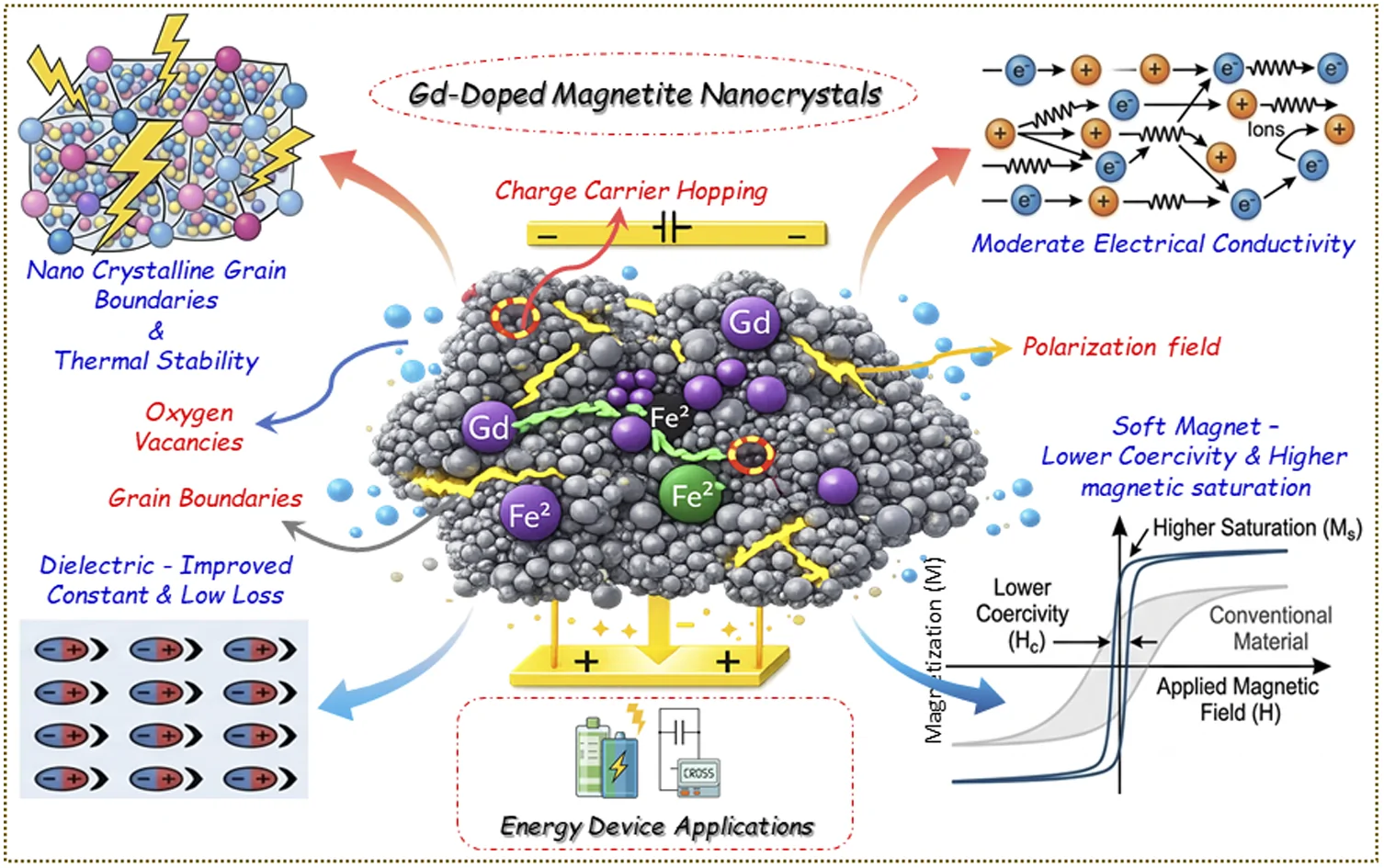

## 1. Introduction

Ferrites are a class of materials that exhibit a wide range of electrical, magnetic, and dielectric properties, making them highly suitable for diverse scientific and technological applications.^1,2^ These materials have been extensively utilized in electronics, optoelectronics, magnetics, magneto-electronics, electrochemical systems, and biotechnology due to their remarkable functional versatility.^3–5^ Specifically, the electrical behavior of magnetic materials is important for their applications in electronic devices.^6–9^ The spinel ferrites NiFe_2_O_4_, MnFe_2_O_4_, and ZnFe_2_O_4_ exhibit higher saturation magnetization and permeability at higher frequencies, as well as relatively higher electrical resistivity, thereby promoting their use in solid-state electronic and magneto-electronic systems.^10–12^ These properties of ferrites help in the fabrication of advanced devices for controlled charge transport.

Magnetite (Fe_3_O_4_) is interesting among other spinel ferrites due to its inverse spinel structure, which provides both Fe^2+^ and Fe^3+^ ions and enables hopping of electrons in the octahedral (B) sites.^13,14^ This feature greatly enhances the material's electrical conductivity and improves dielectric polarization. The general formula of spinel ferrites is MFe_2_O_4_, where M metal ions are found at the tetrahedral (A) and octahedral (B) sites of the FCC oxygen lattice, and their distribution closely relates to the physical properties.^15^ The dielectric response of Fe_3_O_4_ is typically governed by Maxwell–Wagner interfacial polarization and electron hopping mechanisms, leading to high permittivity at lower frequencies. Despite these advantages, Fe_3_O_4_ nanocrystals suffer from limitations including relatively high dielectric loss, moderate electrical conductivity, particle agglomeration, and limited control over charge-carrier dynamics, which adversely affect dielectric performance and energy storage efficiency. Nanoscale Fe_3_O_4_ has a higher surface-to-volume ratio and a tunable microstructure, which can favorably affect its dielectric behavior, electrical conductivity, and interfacial polarization, and hence have a considerable impact on the design of energy storage and electronic devices.^16^ The dielectric response of Fe_3_O_4_ is more often than not described in terms of the Maxwell–Wagner interfacial polarization and hopping of electrons. It is associated with higher dielectric permittivity at lower frequencies. Fe_3_O_4_ nanocrystals are associated with significant drawbacks, including higher dielectric loss, moderate electrical conductivity, particle agglomeration, and a lack of control over charge-carrier dynamics, thereby adversely affecting overall dielectric performance and energy storage efficiency.^17^ In a bid to address some of the foregoing drawbacks, cation substitution has been adopted as a desirable instrumental approach for modifying the electrical and magnetic properties of ferrites. Recent studies have aimed to improve ferrites' properties *via* substitution (doping) strategies. Doping a ferrite can modulate lattice strain, thereby selectively altering cation distribution and influencing magnetic interactions. This, in turn, affords some control over dielectric polarization, as well as conductivity, magnetization and anisotropy. Previous studies have shown that rare-earth doping has emerged as an effective approach to tailor ferrite characteristics to enhance its potential for next-generation applications.^18–20^ Ynasfi *et al.* investigated La^3+^ substitution in manganese ferrites (MnLa_*x*_Fe_2−*x*_O_4_) and reported a decrease in lattice parameters,^21^ and similar variations in results have been reported by Z. A. Gilani *et al.* for Nd-doped Li–Ni nano-ferrites (LiNi_0.5_Nd_*x*_Fe_2−*x*_O_4_).^22^ Moreover, T. Kambre *et al.* reported enhanced dielectric behavior in yttrium-doped hematite,^23^ and T. Kamakshi *et al.* observed changes in the structural, morphological, and optical properties of Fe_3_O_4_ nanoparticles as a result of Ni doping.^24^ Furthermore, M. Aghazadeh *et al.* reported improved dielectric properties in Sm^3+^-doped Fe_3_O_4_ nanoparticles.^25^ Also, A. Neto *et al.* synthesized Fe_3_O_4_ doped with Ce^4+^, Co^2+^, Mn^2+^, and Ni^2+^*via* co-precipitation,^26^ while M. Zeng *et al.* prepared rare-earth-doped Fe_3_O_4_ nanoparticles (La^3+^, Ce^3+^, Dy^3+^)^27^ using a hydrothermal method, highlighting the effectiveness of doping in tailoring ferrite properties.

The rare-earth ion Gd^3+^ has attracted considerable attention due to its large ionic radius, high magnetic moment, high spin (S_Gd_^3+^ = 7/2) and unique half-filled 4f^7^ electronic configuration.^28,29^ Even a small quantity of Gd can affect the ferrite properties. Several studies report that Gd incorporation significantly modifies the structural, magnetic, and electrical behavior of ferrite systems. Gd-doped NiFe_2_O_4_,^30^ Gd-doped BaTiO_3_ (ref. 31) and Zn–Mg nano-spinel ferrites^32^ exhibit improved dielectric properties, while R. A. Pawar *et al.*^33^ reported enhanced functional characteristics in Gd^3+^-substituted Co–Zn ferrites. Similarly, C. V. Ramana *et al.*^3^ confirmed improved electrical conductivity in Gd-doped cobalt ferrites, highlighting the role of rare-earth ions in tuning charge transport. R. Bavandpour *et al.*^34^ also reported improved performance in modified CuFe_2_O_4_ nanoparticles. Furthermore, Sun *et al.*^35^ showed that La- and Gd-doping in Ni–Zn ferrites enhances high-frequency dielectric properties, confirming the effectiveness of rare-earth doping for improving polarization mechanisms. It has also been reported that Gd doping can improve dielectric properties, increase resistivity, and reduce dielectric and magnetic losses, although excessive doping may lead to structural disorder and reduced magnetic interactions.

In this context, the present work reports a systematic investigation of Gd-doped Fe_3_O_4_ nanocrystals synthesized *via* the co-precipitation method. The dopant Gd concentration was taken as 0 ≤ *x* ≤ 0.05 to systematically investigate its influence on the structural, dielectric, electrical and magnetic properties of Fe_3_O_4_ while maintaining the characteristic spinel framework. However, very limited literature is available on Gd-doped Fe_3_O_4_, and the reported studies are mainly confined to isolated investigations of structural, magnetic, and dielectric properties, lacking a comprehensive and correlated analysis. Thus, this work establishes a strong correlation between structure, defect chemistry, dielectric response, electrical conductivity, and magnetic behavior. The results highlight systematic modification of dielectric polarization and charge transport through controlled Gd substitution, thereby underscoring the potential of these materials for multifunctional electronic device applications.

## 2. Experimental details

Gd-doped magnetite nanocrystals were synthesized *via* a co-precipitation route in an aqueous medium, as shown in Scheme 1. The precipitation was carried out by the controlled addition of NH_4_OH (pH ≈ 11), with PEG-600 employed as a capping agent to regulate particle growth and prevent oxidation. The obtained precipitate was magnetically separated, thoroughly washed, and vacuum-dried. A series of samples (GdF0, GdF1, GdF2 & GdF3) with varying Gd concentrations (*x* = 0.000, 0.015, 0.025, and 0.050) were prepared. The detailed synthesis procedure is provided in the SI file.

**Scheme 1:**
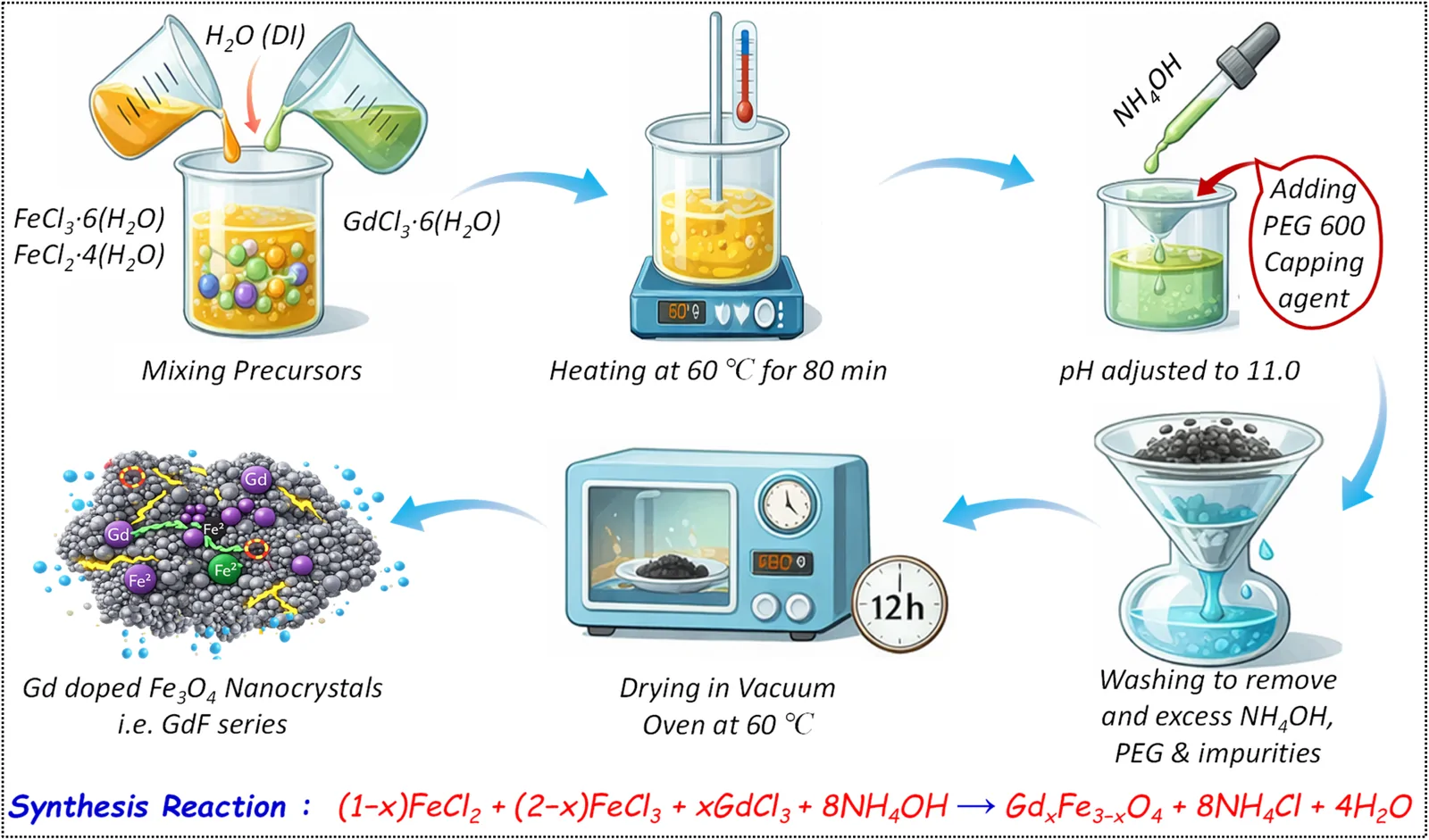
Schematic of the synthesis of Gd-doped Fe_3_O_4_ nanocrystals.

## 3. Results and discussion

The synthesized samples were systematically investigated for their structural, thermal, dielectric, electrical, morphological, and magnetic properties using various characterization techniques; details are provided in the SI file. The corresponding results obtained from these techniques are discussed in the following sections.

### 3.1. X-ray diffraction analysis

The investigation of the Gd-substituted samples for phase formation and structural evolution was conducted using XRD at room temperature. XRD patterns of all samples are shown in Fig. 1. All the obtained diffraction peaks were indexed to the cubic inverse spinel structure of Fe_3_O_4_ with the space group *Fd*3̄*m*, confirming that the essential cubic crystal symmetry of magnetite is retained after Gd incorporation.^36^ The observed reflections around 2*θ* ≈ 30°, 35°, 43°, 54°, 57°, and 63° were indexed to (220), (311), (400), (422), (511) and (440) crystallographic planes, respectively, and were found to be consistent with the available JCPDS data of magnetite. This confirms the synthesis of a single-phase spinel ferrite with no impurity phases. The lack of additional peaks associated with Gd_2_O_3_ or any other iron oxide polymorphs suggests that the incorporation of Gd^3+^ ions into the Fe_3_O_4_ lattice occurs without the formation of a distinct phase.

**Fig. 1:**
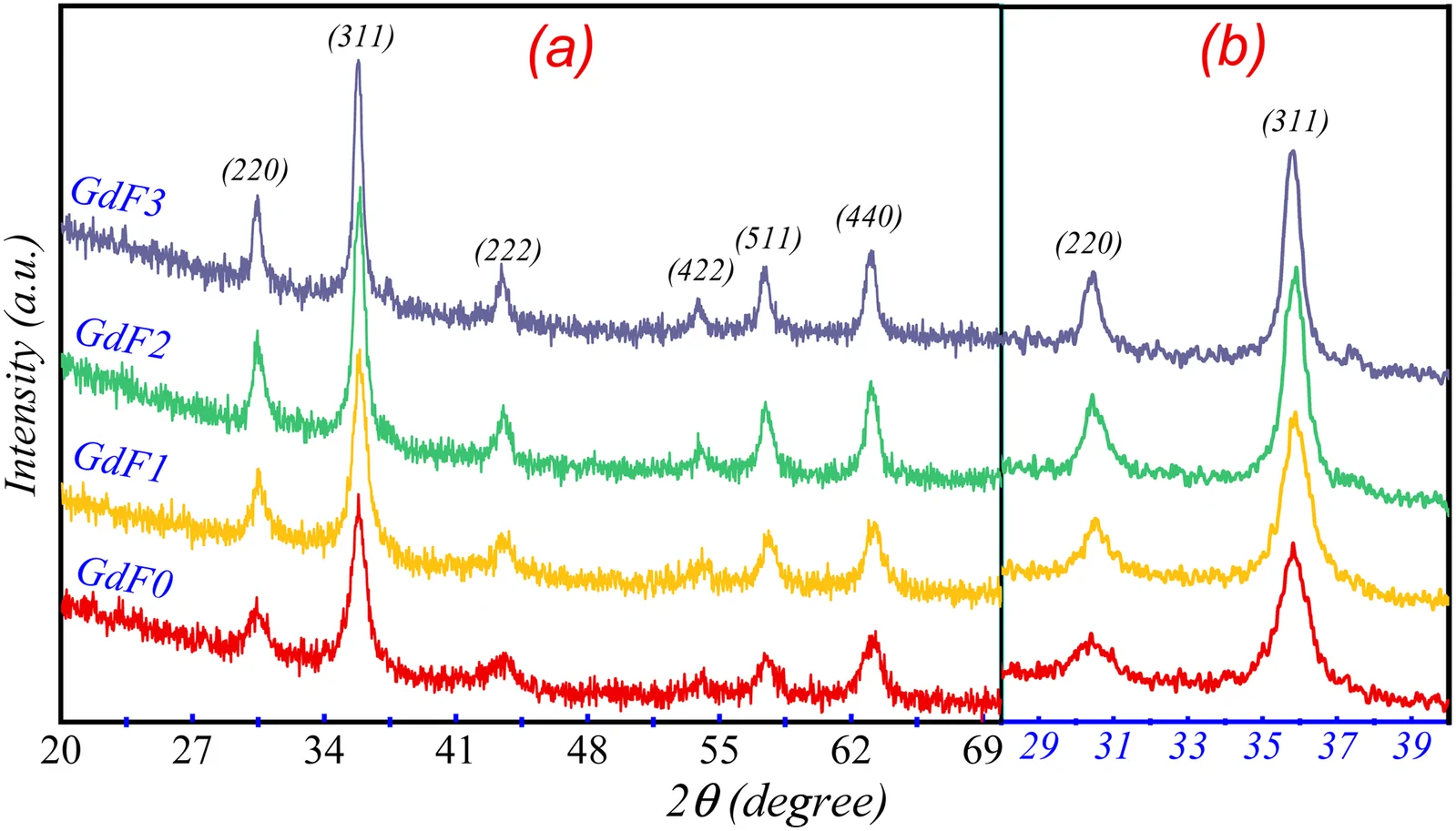
X-ray diffraction in the (a) broad theta range and (b) specific range of the cubic crystal structure of the GdF samples.

The diffraction patterns show that as Gd content increased, the Bragg peaks shifted to lower diffraction angles. This reflects the expansion of the lattice, which occurs as Fe^3+^ ions (ionic radius ≈ 0.645 Å and octahedral) are replaced by Gd^3+^ ions (≈0.938 Å).^37^ The lattice parameter ‘*a*’ for each sample was calculated using Bragg's law for the cubic system (eqn (1)).1
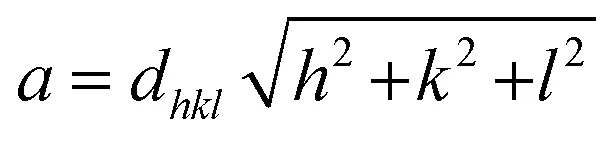
where ‘*d*’ is the interplanar spacing and (*hkl*) are the Miller indices of the corresponding reflection. The calculated lattice parameter shows a gradual increase (from 8.3484 to 8.3665 Å) with Gd concentration.^38^ Although Gd substitution expands the lattice because of the larger ionic radius of Gd^3+^, the microstrain decreases, suggesting partial relaxation of long-range lattice strain. At the local scale, however, the ionic-size mismatch can modify metal–oxygen bond environments and introduce local structural heterogeneity. This trend is consistent with Vegard's law and indicates successful substitutional incorporation of rare-earth ions into the spinel lattice.^39^

The inverse spinel structure of magnetite can be represented as^36^ (Fe^3+^)_A_(Fe^3+^ Fe^2+^)_B_O_4_, where tetrahedral (A) sites are occupied by Fe^3+^ ions and octahedral (B) sites contain both Fe^3+^ and Fe^2+^ ions.^40^ Due to their larger ionic radius and preference for higher coordination, Gd^3+^ ions may preferentially occupy the octahedral sites, causing a redistribution of Fe^3+^ ions between the A and B sites. This cation redistribution induces local lattice distortion and affects electron hopping between Fe^2+^ and Fe^3+^ ions at octahedral sites, which is crucial for electrical conduction and dielectric polarization. Therefore, the structural changes observed in the XRD patterns are expected to strongly influence the dielectric and electrical behavior of the present samples.

The various structural parameters obtained from the Rietveld refinement of this data are given in the SI (Table S1). The average crystallite size ‘*D*’ was estimated from the most intense (311) reflection using the Debye–Scherrer relation (eqn (2)).^41^2
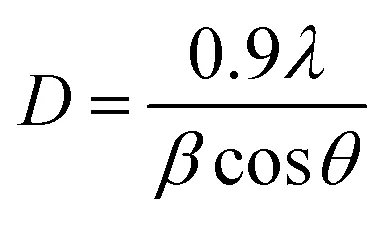
where *λ* is the X-ray wavelength, *β* is the full width at half maximum (FWHM), and *θ* is the Bragg angle. The calculated crystallite size lies in the 7.24–14.81 nm regime and exhibits a slight increase with Gd concentration. This increase may be attributed to lattice expansion and reduced internal strain resulting from the substitution of larger-sized Gd^3+^ ions, thereby facilitating grain growth during synthesis. The nanoscale crystallite size ensures a high surface-to-volume ratio and a significant grain-boundary contribution, both of which are important for dielectric polarization and charge-transport mechanisms in ferrite-based devices.

The microstrain (*ε*) and dislocation density (*δ*) were also estimated using standard relations given by eqn (3) and (4), respectively.3
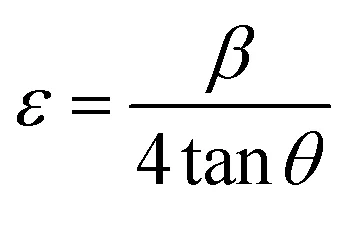
4
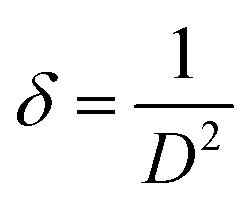


The calculated microstrain decreases slightly with increasing Gd content, indicating partial relaxation of lattice distortion despite the larger ionic radius of Gd^3+^. This behavior suggests that Gd substitution reduces internal defects and stabilizes the crystal structure. The decrease in dislocation density with increasing crystallite size further supports the improvement in crystallinity of doped samples. The unit cell volume (*V* = *a*^3^) increases with Gd incorporation, which directly influences the hopping length between magnetic ions at tetrahedral and octahedral sites. The hopping lengths for A- and B-sites in spinel ferrites can be expressed as (eqn (5) and (6)), respectively.^42^5
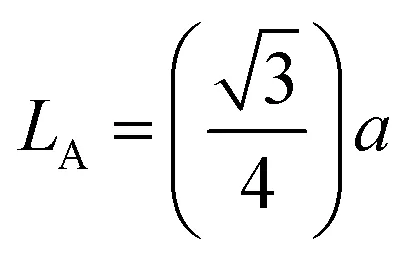
6
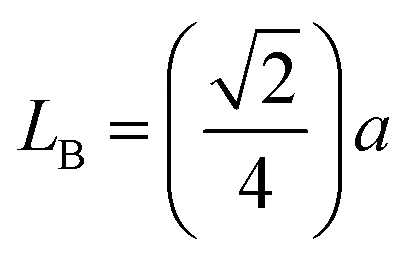


Electrical conduction occurs primarily *via* electron hopping between Fe^2+^ and Fe3^+^ ions at octahedral sites; any change in the hopping length directly affects electrical resistivity and dielectric relaxation behavior. So, the structural expansion induced by Gd incorporation is expected to reduce hopping probability and modify the dielectric dispersion characteristics of the samples. The X-ray density (*ρ*_x_) was calculated using eqn (7).7
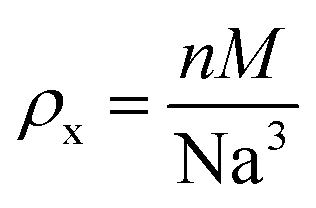
where *M* is the molecular weight, and *N*_a_ is Avogadro's number. The density increases with Gd content due to Gd's higher atomic mass relative to Fe. Increased density and improved crystallinity are favorable for enhanced dielectric stability and reduced porosity-related losses. Thus, XRD analysis confirms the formation of single-phase Gd-doped magnetite with a cubic inverse spinel structure. The systematic variation in lattice parameter, crystallite size, microstrain and unit-cell volume with Gd concentration indicates successful substitution of Gd^3+^ into the Fe_3_O_4_ lattice and consequent modification of the structural parameters. These structural changes are expected to play a decisive role in governing the thermal stability, dielectric polarization and electrical transport behavior of the present nanocrystalline ferrites, thereby influencing their suitability for energy-related device applications.

### 3.2. Thermal analysis

The TGA of the Gd-doped Fe_3_O_4_ samples presents a detailed account of the system's thermal stability and integrity and the effect on the phases within the system. The Gd-substituted magnetite nanocrystals' thermal stability and decomposition behavior were analyzed *via* simultaneous TG and DSC in the range of room temperature to 1100 °C. The combined weight-loss and heat-flow curves for all compositions are shown in Fig. 2. All samples exhibit a multi-step weight-loss behavior, indicating removal of physically adsorbed species, decomposition of residual precursors, and structural relaxation processes. Between room temperature and ∼150 °C, nearly all samples exhibited small weight loss. This weight loss results from the desorption of moisture physically adsorbed on the sample and the loss of surface-bound volatile species. This behavior is common in oxides and ferrites synthesized *via* wet chemical or solid-state methods.^43^ This weight loss indicates decreased surface adsorption for lower-Gd compositions. This behavior may be due to reduced porosity and surface-site coverage at lower Gd compositions. This stage is more pronounced for the GdF0 sample, indicating a higher surface adsorption capability due to its smaller crystallite size and larger surface area.

**Fig. 2:**
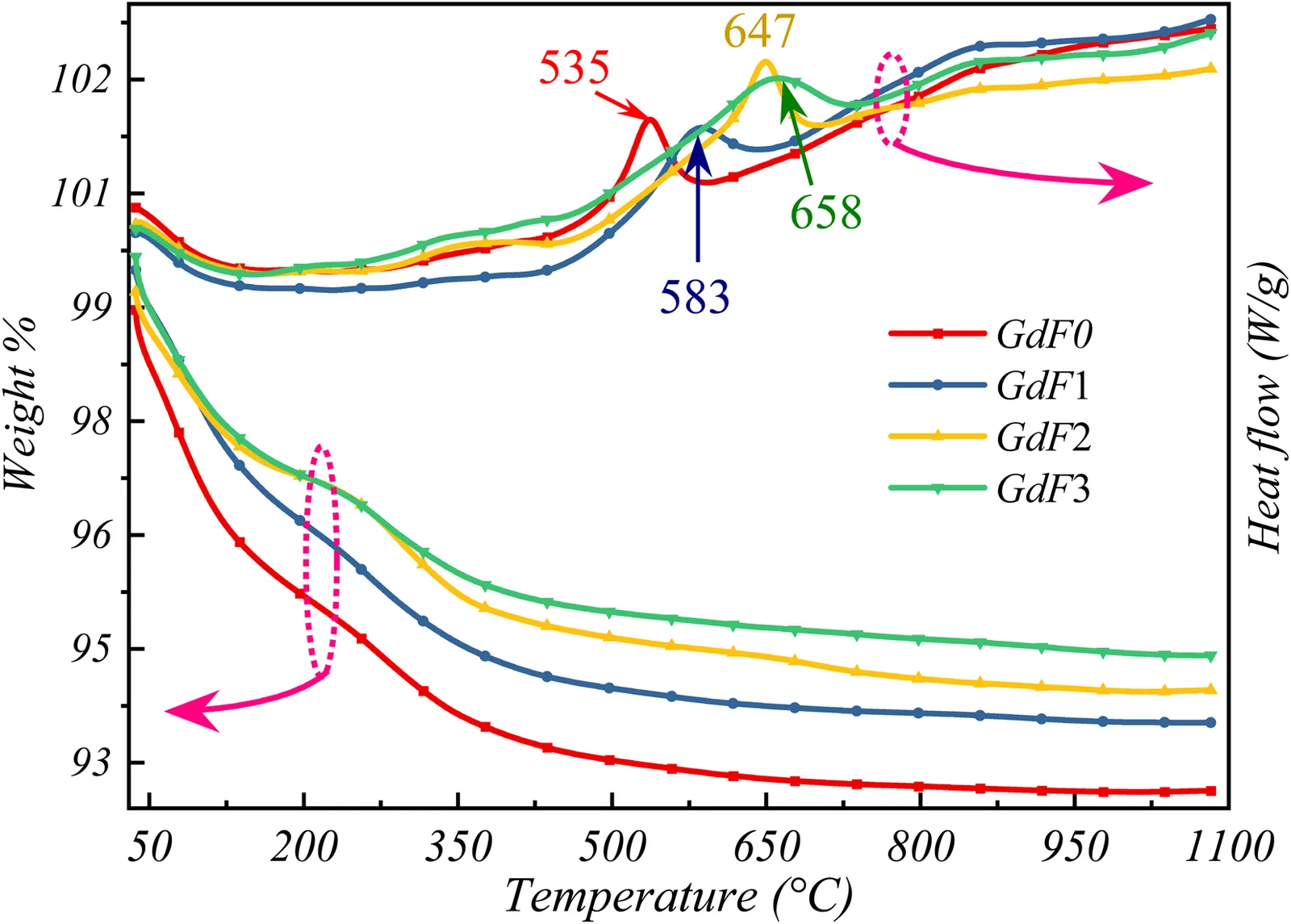
Weight-loss curves and heat-flow patterns with temperature of the GdF samples.

A second gradual weight-loss region, extending from approximately 150 °C to 400 °C, is associated with the removal of residual organic species (such as PEG or hydroxyl groups) and the decomposition of surface-bound intermediates originating from the synthesis route. The presence of rare-earth Gd^3+^ ions appears to slightly suppress the magnitude of this weight loss, suggesting improved thermal stability of the doped samples. The reduction in total mass loss with increasing Gd concentration indicates that Gd incorporation enhances structural compactness and reduces surface defects, thereby lowering the amount of loosely bound species. Beyond ∼400 °C, the weight-loss curves tend to stabilize, indicating that the ferrite structure becomes thermally stable and no major decomposition occurs at higher temperatures. The residual mass at high temperature is higher for Gd-substituted samples, which is consistent with the higher atomic mass of gadolinium and the improved structural integrity of doped ferrites. Overall, the total weight loss decreases with increasing Gd concentration, confirming enhanced thermal robustness upon Gd substitution. From the TG analysis, it can also be determined that Gd-doped magnetite exhibited improved thermal stabilization as the Gd-ion doping increased.^44^

The stable mass plateau observed above ∼800 °C indicates that no significant further mass loss occurs, suggesting enhanced thermal stability of the final solid phase. Moreover, improved thermal stability was also observed to correlate with increased Gd concentrations. The substitution of Gd^3+^ ions modifies the local metal–oxygen bonding environment, leading to changes in bond lengths and increased local structural heterogeneity. This would also result in lower lattice energy and slower diffusion in the system during the thermal treatment. Thus, any oxygen-related defects present are likely due to thermal treatment, surface effects, or non-stoichiometry.

The exothermic peak observed in the DSC curve is attributed to crystallinity enhancement and grain growth resulting from atomic rearrangement and structural ordering. Additionally, thermal energy facilitates redistribution of Fe^2+^ and Fe^3+^ ions between tetrahedral and octahedral sites toward an equilibrium configuration. The relaxation of lattice strain induced by the substitution of larger Gd^3+^ ions and the annihilation of structural and oxygen-related defects also contribute to the release of heat during this process. The DSC curve exhibits strong exothermic peaks at 535 °C, 583 °C, 647 °C, and 658 °C, shifting to higher temperatures with increasing concentration. The exothermic peak is reported to be associated with the formation of the α-Fe_2_O_3_ phase.^45^ The heat evolved in this exothermic process is attributed to enhanced crystallinity and grain growth, reordering of Fe^2+^ and Fe^3+^ ions, and relaxation of lattice strain introduced by larger Gd^3+^ ions. It is reported that doping with rare-earth or transition-metal ions in ceramics introduces lattice defects such as oxygen vacancies, cation vacancies, and oxygen interstitials within the host lattice. These defects are reported to optimize multifunctional properties of significant technological interest.^46^

The DSC curves provide further insight into the thermal events occurring during heating. A broad endothermic feature observed at lower temperatures corresponds to dehydration and removal of adsorbed species. At intermediate temperatures, distinct exothermic peaks are visible, marked in Fig. 2 around 535 °C, 583 °C, 647 °C, and 658 °C for different compositions. These thermal events are associated with crystallite growth, phase stabilization, and ordering within the spinel lattice. The slight shift in the peak positions with Gd content suggests that the substitution of larger Gd^3+^ ions alters the lattice energy and influences the crystallization dynamics. In particular, the movement of exothermic peaks toward higher temperatures for higher Gd content indicates stronger lattice distortion, enhanced thermal stability and delayed structural rearrangement, which can be attributed to stronger metal–oxygen bonding and lattice distortion introduced by Gd^3+^ ions.^46^ The exothermic peaks observed at 535 °C, 583 °C, 647 °C, and 658 °C are attributed to thermally activated structural ordering processes, including defect annealing, lattice relaxation, and crystallite growth in Gd-doped spinel Fe_3_O_4_. The suppression of large exothermic transitions suggests that most of the structural ordering is already achieved during synthesis, and Gd incorporation stabilizes the inverse spinel framework against further transformation.

At higher temperatures, the broad exothermic background indicates continued grain coarsening, enhanced stabilization of the spinel phase, and a possible oxidation-induced phase transformation of magnetite toward maghemite or hematite (Fe_3_O_4_ → α-Fe_2_O_3_). Rare-earth substitution (Gd^3+^) introduces lattice strain and defect centers due to ionic size mismatch, thereby increasing the activation energy for structural ordering and shifting thermal events to higher temperatures. These interpretations are consistent with reported thermal analysis studies of doped spinel ferrites and oxide materials. The observed improvement in thermal stability is highly beneficial for dielectric and electrical applications where materials are subjected to elevated temperatures.

### 3.3. FT-IR analysis

FTIR was also used to study the Gd-substituted magnetite systems by investigating the resulting changes in local bonding and the framework. The mid-infrared region (400–4000 cm^−1^) of the GdF0, GdF1, GdF2, and GdF3 samples is shown in the FTIR spectra in Fig. 3. All samples exhibit the characteristic absorption spectra of spinel ferrites. Hence, the presence of the inverse spinel Fe_3_O_4_ structure after Gd substitution is confirmed. In general, spinel ferrites consist of two prominent metal–oxygen vibration bands. The first corresponds to the tetrahedral (A-site) metal–oxygen bonds, and the second corresponds to the octahedral (B-site) metal–oxygen bonds. The first band (*ν*_1_) is the intrinsic stretching vibration of a metal–oxygen bond and is observed in the range of ∼550–600 cm^−1^. The second band (*ν*_2_) is an octahedral metal–oxygen vibration and appears in the range of ∼400–450 cm^−1^.^47^

**Fig. 3:**
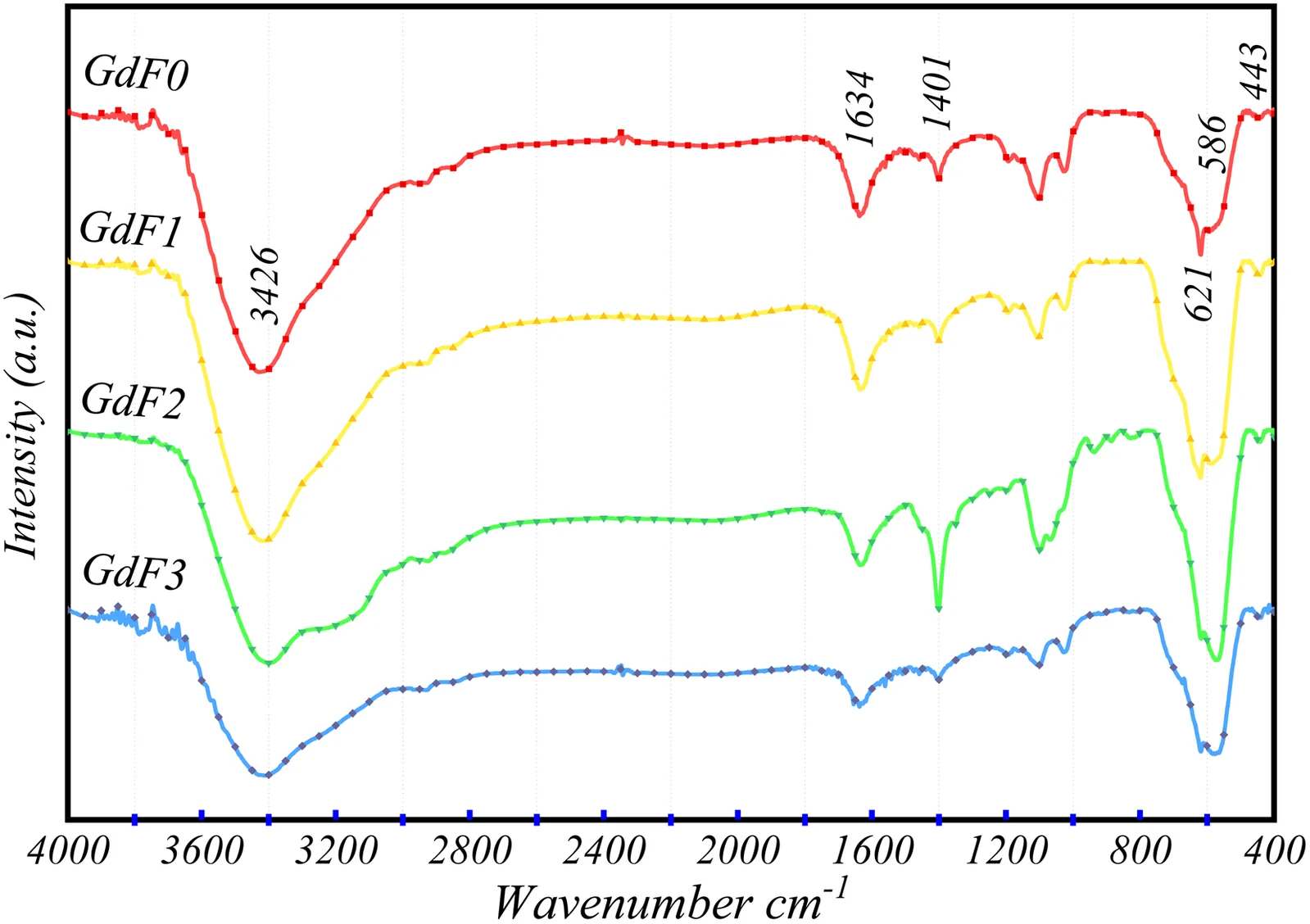
FT-IR analysis of the Gd_*x*_Fe_3−*x*_O_4_ composites.

In the present spectra, these two fundamental absorption bands are clearly visible for all compositions. The band in the higher wavenumber region corresponds to the Fe–O stretching vibration at tetrahedral sites, while the lower-frequency band arises from octahedral Fe–O/Gd–O vibrations. The presence of these two distinct bands confirms that the spinel ferrite structure remains intact after incorporation of Gd^3+^ ions. However, careful examination reveals slight shifts and broadening of the octahedral-site band with increasing Gd content.^48^ This behavior can be attributed to the substitution of larger-sized Gd^3+^ ions at octahedral sites, which alters the bond lengths and local symmetry at these sites. Because Gd^3+^ ions preferentially occupy octahedral positions in the spinel lattice, their incorporation leads to increased lattice distortion and modification of the metal–oxygen bond strength. Consequently, the octahedral vibration band broadens and shifts slightly, indicating increased structural disorder and local strain. Additional absorption features observed at higher wavenumbers are associated with surface and environmental effects. For all samples, a band in the region of ∼3400 cm^−1^ is associated with the O–H stretching vibration of water and surface hydroxyl groups. The band in the region of ∼1600–1650 cm^−1^ is associated with the bending of the absorbed water. The bands in the region of ∼1000–1400 cm^−1^ are the residual carbonates and/or the organic groups associated with the synthesis. The gradual reduction of these bands with increasing Gd content is associated with improved crystallinity and decreased surface impurities in the samples.

The shift in vibrational frequencies with increasing Gd concentration can be correlated with changes in bond length and force constants. Substitution by heavier Gd^3+^ ions increases the average metal–oxygen bond length and reduces vibrational frequency at octahedral sites. At the same time, the redistribution of Fe ions between tetrahedral and octahedral sites modifies the force constants of Fe–O bonds, leading to subtle changes in the position of the tetrahedral band. Such modifications to local bonding environments are known to influence dielectric polarization mechanisms and electron-hopping processes in ferrites. Since electrical conduction in magnetite is governed by electron hopping between Fe^2+^ and Fe^3+^ ions at octahedral sites, any change in bond length or lattice distortion can significantly affect dielectric relaxation and conductivity. It is also observed that as Gd content increases, the overall band intensity becomes slightly broader and less sharp, indicating increased local structural heterogeneity around the metal–oxygen bonds.^49^ This behavior is typical of rare-earth-substituted ferrites, where a large ionic-size mismatch introduces local strain and modifies the vibrational landscape of the lattice. Such structural distortion can enhance interfacial polarization and contribute to dielectric dispersion at lower frequencies. Therefore, the FTIR results corroborate the XRD and thermal analyses by confirming the formation of a stable inverse spinel structure with successful incorporation of Gd^3+^ ions into the Fe_3_O_4_ lattice.

### 3.4. X-ray photoelectron spectroscopy analysis

The surface chemical composition and oxidation states of the elements present in undoped (GdF0) and Gd-doped (GdF3) samples were investigated using XPS, as shown in the survey spectra (Fig. 4). The spectra confirm the presence of Fe, O, Gd, and a small amount of adventitious carbon. The peak at ∼284.8 eV corresponds to the C_1s_ signal, which originates from surface contamination and is commonly used for charge correction. The Fe_2p_ spectrum exhibits two main peaks corresponding to Fe 2p_3/2_ and Fe 2p_1/2_ located at binding energies around ∼709–712 eV and ∼725 eV,^50^ respectively, with a spin–orbit splitting of approximately 13–14 eV. The Fe 2p_3/2_ peak reveals contributions from both Fe^2+^ and Fe^3+^ oxidation states, showing the mixed-valence nature of Fe ions characteristic of the magnetite structure. The Fe^2+^ component typically appears near ∼709.5 eV, while the Fe^3+^ component is centered around ∼711 eV.^51^ The coexistence of Fe^2+^ and Fe^3+^ ions is a distinctive feature of Fe_3_O_4_ and is responsible for electron hopping between octahedral B sites, which significantly influences the electrical and dielectric properties.

**Fig. 4:**
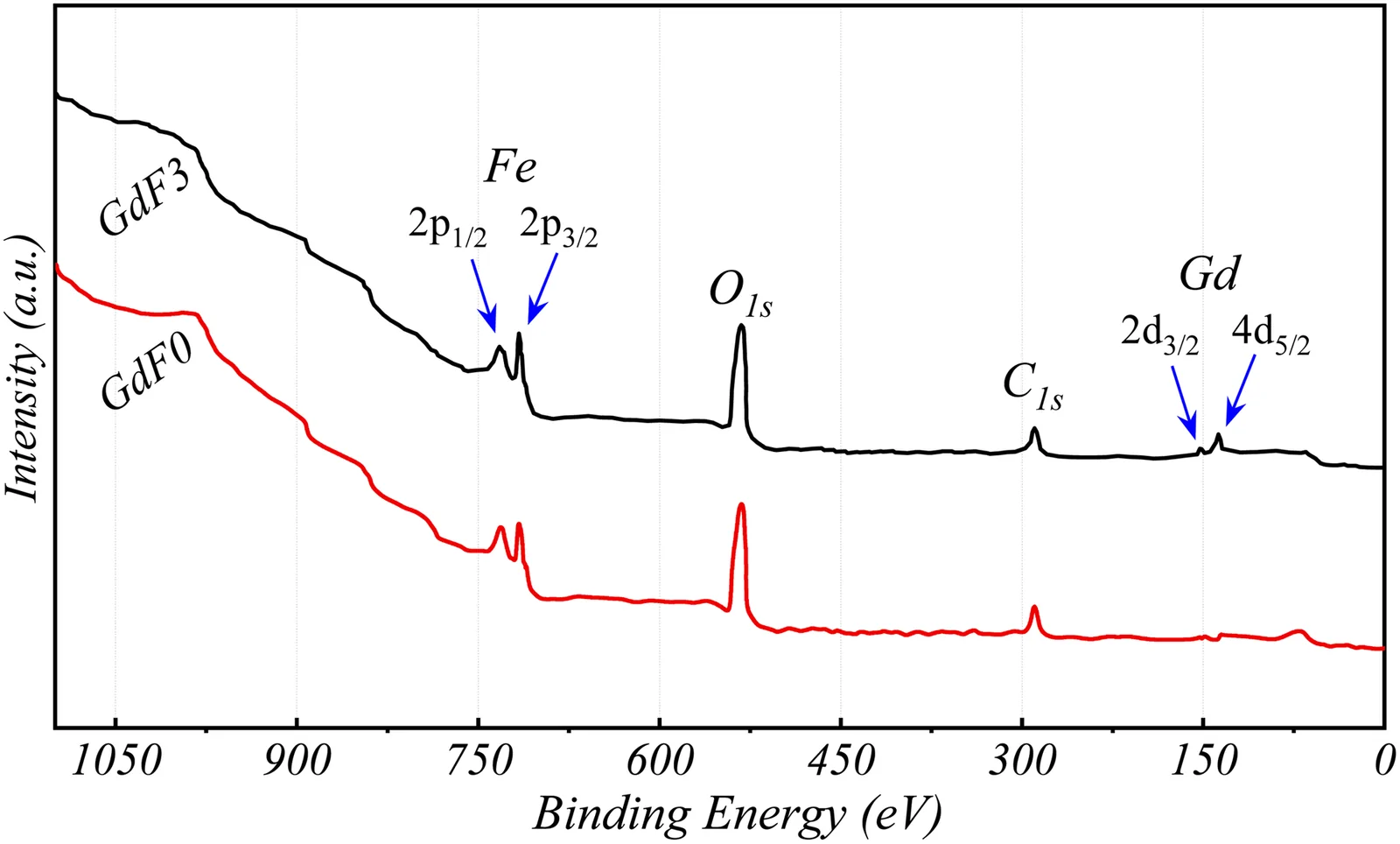
XPS analysis of GdF0 and GdF3.

The O 1s spectrum, as shown in Fig. 4 (around 529–531 eV), comprises two main components. One peak is observed at a lower energy of ∼529.5 eV, corresponding to lattice oxygen (O^2−^) bonded to Fe in the spinel Fe–O/Gd–O framework. A second peak observed at ∼531 eV is attributed to surface-adsorbed oxygen species or defect-related oxygen environments. For the Gd-doped GdF3 samples, additional peaks corresponding to Gd core levels are observed, confirming the successful incorporation of Gd ions into the Fe_3_O_4_ lattice. The Gd 4d peaks generally appear in the range of ∼142–148 eV. These peaks are characteristic of Gd^3+^ oxidation states and indicate that Gd ions substitute Fe^3+^ sites in the spinel lattice without forming separate secondary phases. The introduction of Gd^3+^ ions in place of octahedral-sited Fe^3+^/Fe^2+^ ions results in lattice distortion and local structural disorder due to the ionic size mismatch. These defects modify the local electronic structure and influence the Fe^2+^/Fe^3+^ ratio, thereby affecting electron hopping between octahedral sites. The presence of such defects and mixed-valence states plays a significant role in determining the electrical conductivity, dielectric response, and polarization mechanisms of Gd-doped Fe_3_O_4_.

### 3.5. FESEM analysis

Important insights into the particle morphology, agglomeration behavior and evolution of grain structure of the synthesized nanocrystals were obtained by FE-SEM analysis. The micrographs of the samples are presented in Fig. 5. The undoped sample exhibits a highly dense assembly of nearly spherical nanoparticles with noticeable agglomeration. The agglomeration is mainly attributed to the higher surface energy and strong magnetic dipole–dipole interactions between magnetite nanoparticles. The particle/grain sizes were estimated from these micrographs using ImageJ software. The estimated average particle/grain sizes were 9.60, 12.65, 14.15, and 16.90 nm for *x* = 0.000, 0.015, 0.025, and 0.050, respectively (Table S1 and Fig. S1). The particle/grain size increases steadily with increasing Gd content, consistent with the crystallite size estimated from X-ray diffraction. The nanoparticles appear relatively uniform and closely packed, forming clusters that create a porous network structure. Such morphology is commonly observed in nanoscale ferrites synthesized *via* wet-chemical routes, in which particle coalescence occurs during the drying and calcination stages.

**Fig. 5:**
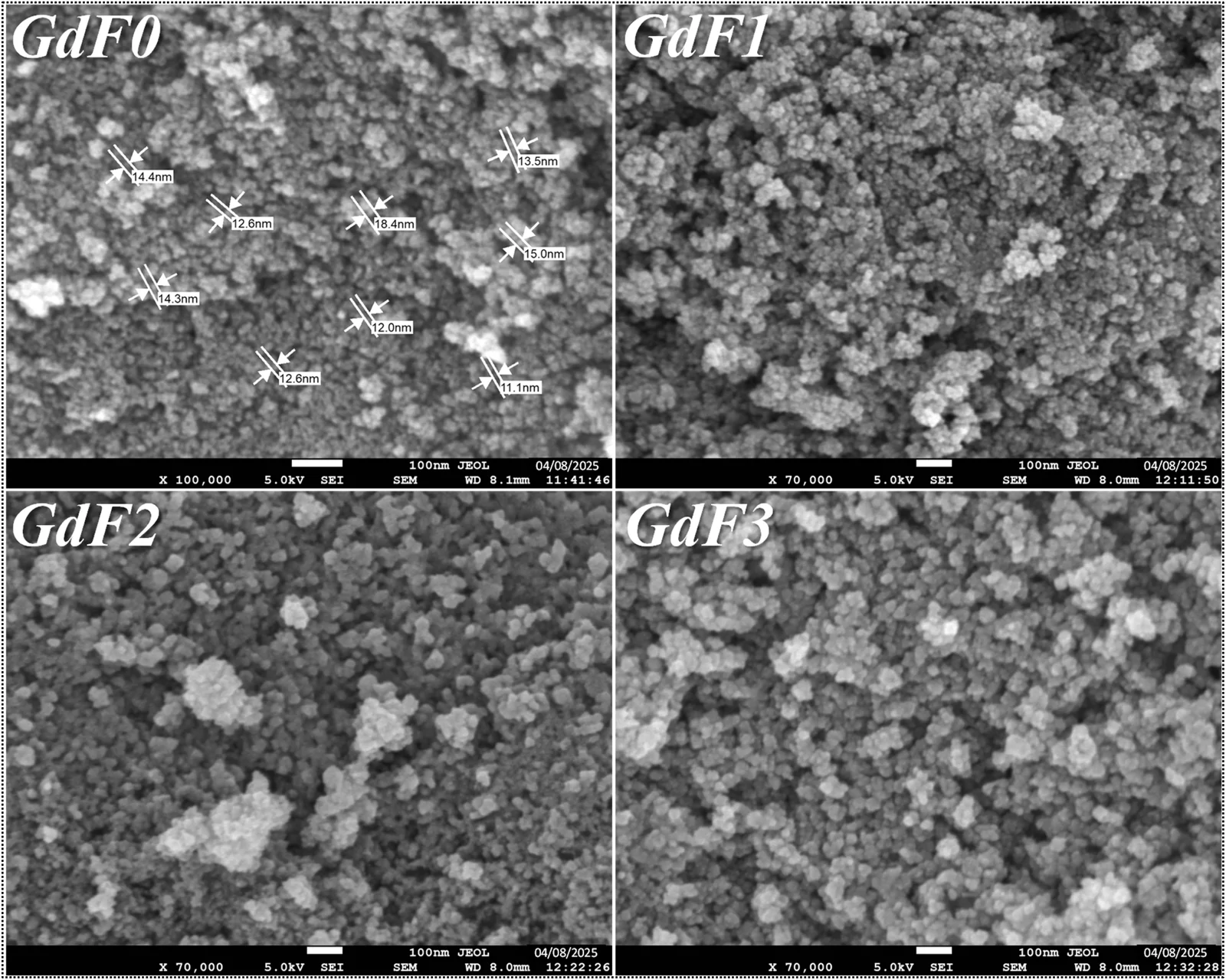
FE-SEM micrographs of the samples.

For the Gd-doped compositions, the FE-SEM micrographs reveal a broadly similar microstructural behavior characterized by densely packed nanosized particles with noticeable agglomeration. The nanoparticles retain a predominantly quasi-spherical morphology and form interconnected clusters that are uniformly distributed across the surface. Compared with the undoped sample, the doped compositions show slightly enhanced particle aggregation, which can be attributed to the incorporation of larger Gd^3+^ ions that modify the nucleation and growth kinetics during synthesis. The presence of Gd ions introduces lattice strain and affects the diffusion processes during crystallization, leading to a marginal increase in grain connectivity and cluster formation. Despite this, the individual grains remain within the nanometer regime and exhibit relatively homogeneous distribution throughout the microstructure. Such agglomerated nanostructures are typical for ferrite nanoparticles due to strong magnetic dipole–dipole interactions and higher surface energy. The formation of interconnected nanoparticle clusters results in a large number of grain boundaries and interfacial regions, which become crucial in ferrite systems, where electrical conduction occurs *via* electron hopping. The presence of such morphologies and microstructures can play a crucial role in charge transport and interfacial polarization mechanisms in ferrite systems.

### 3.6. Dielectric analysis

The dielectric constant characterizes the polarization and charge-transport behavior of Gd-doped Fe_3_O_4_, where Gd^3+^ incorporation induces lattice distortion and structural disorder, which can influence space-charge polarization and charge transport at grain boundaries. The dielectric response of materials exhibits strong frequency and temperature dependence, which can be ascribed to microstructural effects and thermally activated charge carriers. For dielectrics, the complex dielectric constant is expressed as follows:^52,53^

8 ε* (*ω*) = *ε*′(*ω*) − *iε*″(*ω*)

The real part of the dielectric permittivity, *ε*′(*ω*), reflects the material's ability to store and polarize electric charge. The imaginary part, *ε*″(*ω*), reflects the dielectric loss. The temperature dependence of the dielectric constant plays an important role in determining the dielectric and polarization behavior.

Fig. 6 displays *ε*′ as a function of frequency from 30 °C to 160 °C for all the compositions. It shows a higher *ε*′ in the lower-frequency region, followed by a gradual decrease with increasing frequency, stabilizing to an almost constant value in the higher-frequency region. For all four compositions, *ε*′ exhibits the analogous frequency-dependent behavior, which is characteristic of dielectric materials marked by higher ε′ values at lower frequencies that fall and plateau at higher frequencies. For Fe_3_O_4_, this behavior is also influenced by the hopping of electrons between Fe^2+^ and Fe^3+^ ions at octahedral sites and contributes significantly to the dipolar polarization.^54,55^

**Fig. 6:**
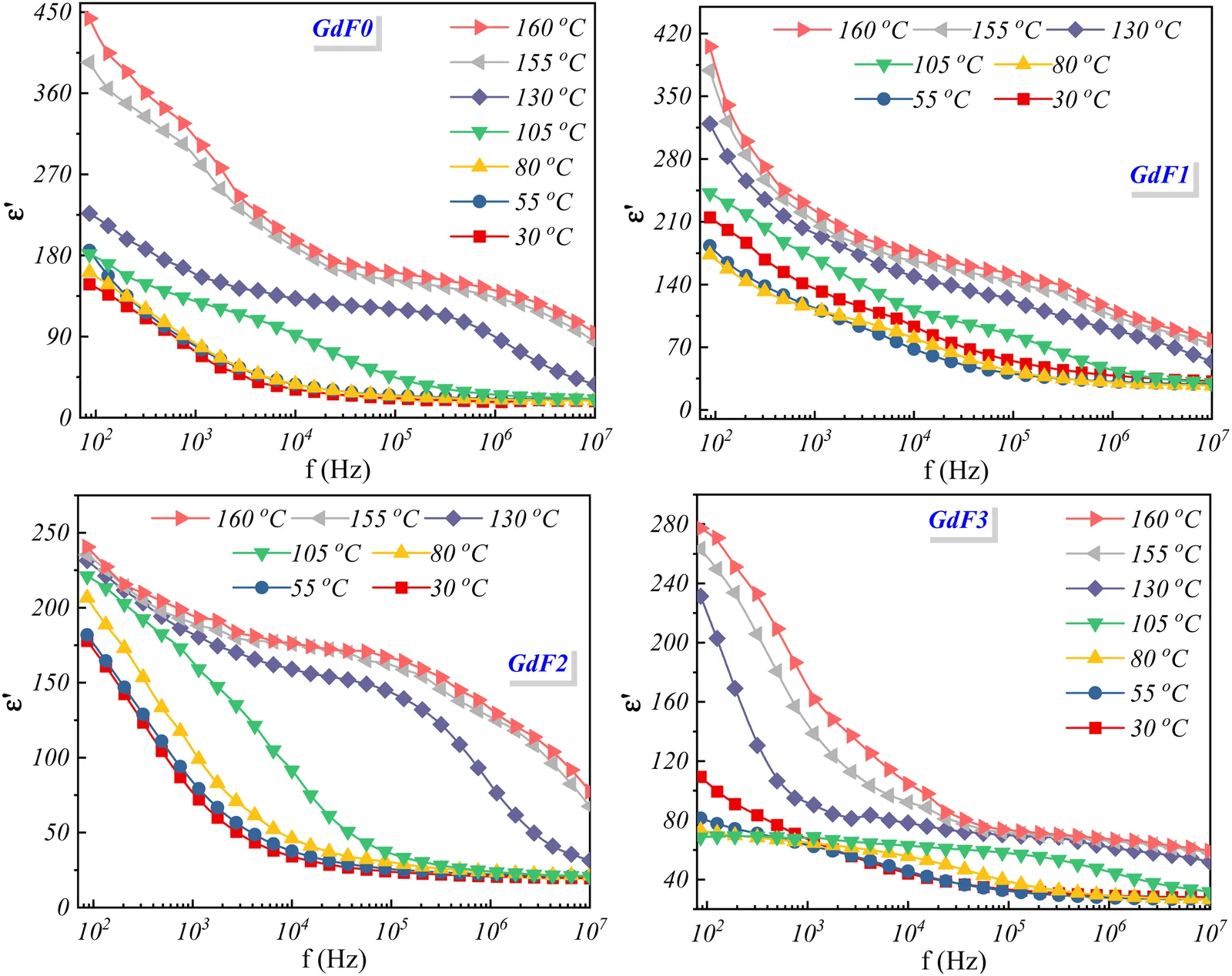
Variation in the real part of the dielectric permittivity as a function of frequency at various temperatures.

Higher *ε*′ is the result of multi-layer capacitor-like, pore-dominated dielectric structures characterized by Maxwell–Wagner polarization at the grain and grain boundary interfaces, as described by Koop's model wherein ferrites are comprised of highly conductive grains and poorly conductive grain boundaries.^56^*ε*′ is higher at lower frequencies because charge carriers accumulate at grain boundaries, creating space charge. Increased frequencies reduce *ε*′ due to the inability of charge carriers and dipoles to keep up with a rapidly changing field.^57^*ε*′ mainly measures the influence of ionic and electronic polarization at higher frequencies and asymptotically approaches the value *ε*_∞_. Numerous studies have shown lower-frequency dielectric enhancement in ferrites, Fe_3_O_4_, and rare-earth-doped ferrites.^58^

The rising temperature leads to an increase in *ε*′ as the mobility of the charge carriers and dipoles, and accordingly the polarization, increases. At lower frequencies, all types of polarization are present, and the rapid rise in dielectric constant with increasing temperature is mainly due to interfacial and dipolar polarizations, which are highly temperature-dependent. At higher frequencies, the temperature dependence becomes insignificant as only the intrinsic polarizability is accounted for in the dielectric constant.^59^ The variations in the magnitude and dispersion of *ε*′ is associated with the increase in Gd content. Upon substituting Gd^3+^ ions into octahedral sites, the Fe^3+^/Fe^2+^ ion sites create lattice defects such as distortion and oxygen vacancies 
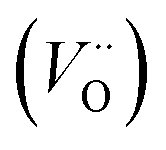
 as a charge compensation mechanism.^60^ These defect states can act as localized polarization centers, strengthening space-charge polarization at lower doping levels. This ion substitution also influences electron hopping between Fe^2+^ and Fe^3+^ ions. These, along with the lattice distortion and defect states, become centers for further polarization, thereby strengthening space-charge polarization and modifying the dielectric response based on Maxwell–Wagner's interfacial polarization and the mechanisms of hopping conduction.

Fig. 7 illustrates the dependence of the imaginary part *ε*″ on the frequency at different temperatures. For all the samples, a typical dielectric relaxation was observed. The values of *ε*″ showed a decreasing tendency as the frequency increased (eqn (9) and (10)).

9 *σ*(*ω*) = *ωε*_0_*ε*″(*ω*)

10 *ε*″(*ω*) ∝ 1/*ω*

where *σ* and *ω* are the electric conductivity and angular frequency, respectively.

**Fig. 7:**
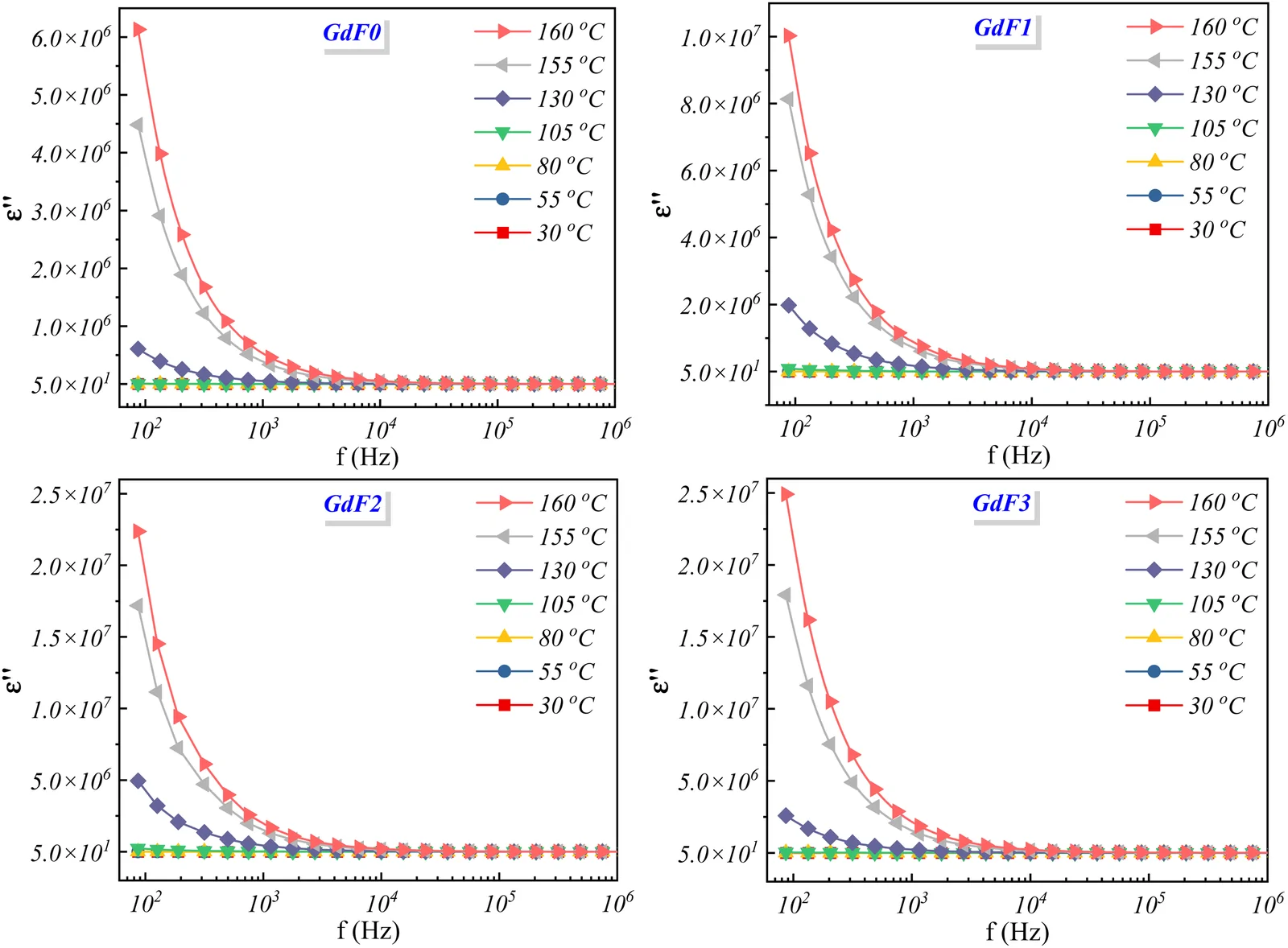
Imaginary part of the dielectric constant *ε*″ as a function of frequency.

The *ε*″ is higher at lower frequencies because of space-charge polarization and the energy dissipated along the grain boundaries. At higher frequencies, *ε*″ decreases as the charge carriers can no longer keep pace with the alternating field, and the polarization of the grains becomes predominant. Further, dielectric loss decreases with increasing Gd concentration^37^ across all compositions and temperatures. The *ε*″ decreases with increasing frequency, indicating that space-charge polarization is the dominant mechanism at lower frequencies. This is indicative of the thermally activated conduction mechanism, which is facilitated by electron hopping between Fe^3+^ and Fe^2+^ ions at the octahedral sites of the spinel lattice, especially in the inverse Fe_3_O_4_.

The dielectric loss (tan *δ*) and quality factor (*Q*) were also assessed to evaluate the overall energy-dissipation capability of the samples. The frequency-dependent variations of tan *δ* (Fig. S2) and *Q* (Fig. S3) for all compositions are presented and discussed in the SI (Sections 2.2 and 2.3). The dielectric loss (tan *δ* = *ε*″/*ε*′) is related to the quality factor as *Q* = 1/tan *δ*. Thus, a lower tanδ and higher *Q* indicate reduced energy dissipation and improved dielectric quality. Both parameters show a dependence on Gd doping concentration, indicating an influence on the polarization and charge-transport processes in Fe_3_O_4_. In addition, both strongly depend on frequency and temperature. The reduction in dielectric loss with increasing Gd concentration can be correlated with changes in the nanocrystal structure. Although dopant Gd^3+^ substitution may introduce local lattice distortion, there is an increase in crystallite size, together with a significant decrease in microstrain and dislocation density. The increase in crystallite size may reduce the relative contribution of grain boundaries, whereas the reduction in microstrain and dislocation density indicates decreased structural disorder and defect-associated charge trapping. Accordingly, the contribution of space-charge & interfacial polarization and associated dielectric dissipation is reduced with increasing Gd content. In addition, Gd incorporation may alter Fe^2+^/Fe^3+^ ratios, thereby influencing charge-carrier hopping and polarization processes.

In Fe_3_O_4_-based systems, dielectric loss is strongly associated with electron hopping between Fe^2+^ and Fe^3+^ at octahedral (B) sites. The substitution of Gd introduces lattice distortion and defect-related structural disorder that can modify space-charge polarization and the hopping pathways of charge carriers at moderate concentrations. At the same time, excessive doping may increase structural disorder and carrier trapping. The broad frequency dispersion and absence of distinct relaxation peaks confirm non-Debye dielectric behavior. It is common to observe heterogeneous conduction and grain boundary-controlled dielectric loss in rare-earth-doped magnetite and spinel ferrites. Since the slope of *ε*″ is less steep at increasing frequencies for the doped samples, this shows that only fast mechanisms of electronic or ionic polarization are present and contribute very little to dielectric loss. Also, the inability of charge carriers to accumulate at grain boundaries reduces *ε*″. With increasing temperature, enhanced hopping conduction accelerates relaxation processes, decreasing relaxation time and shifting relaxation toward higher frequencies. This non-Debye behavior reflects heterogeneous conduction dominated by grain boundaries in Gd-doped Fe_3_O_4_. Therefore, the decrease in dielectric loss is attributed to the combined impact of crystallite growth, reduced lattice disorder, modified grain-boundary contribution, defect chemistry, and thermally activated charge-carrier dynamics in Fe_3_O_4_ samples.

### 3.7. Electrical conductivity analysis

The behavior of electrical conductivity (*ω*) over a wide temperature range is shown in Fig. 8 for each composition. For all compositions, conductivity exhibits the characteristic behavior of a disordered ferrimagnetic oxide, with a plateau at lower frequencies and a rapidly varying region at higher frequencies. Hence, conductivity in the AC sense reflects the dissipation of energy and movement of charge carriers within the sample. In the case of spinel ferrites Fe_3_O_4_, it is stated that electrical conduction is mainly due to the hopping of electrons between Fe^2+^ and Fe^3+^ ions at octahedral sites and is greatly dependent on the presence of defects, grain boundaries, and impurities.^61^

**Fig. 8:**
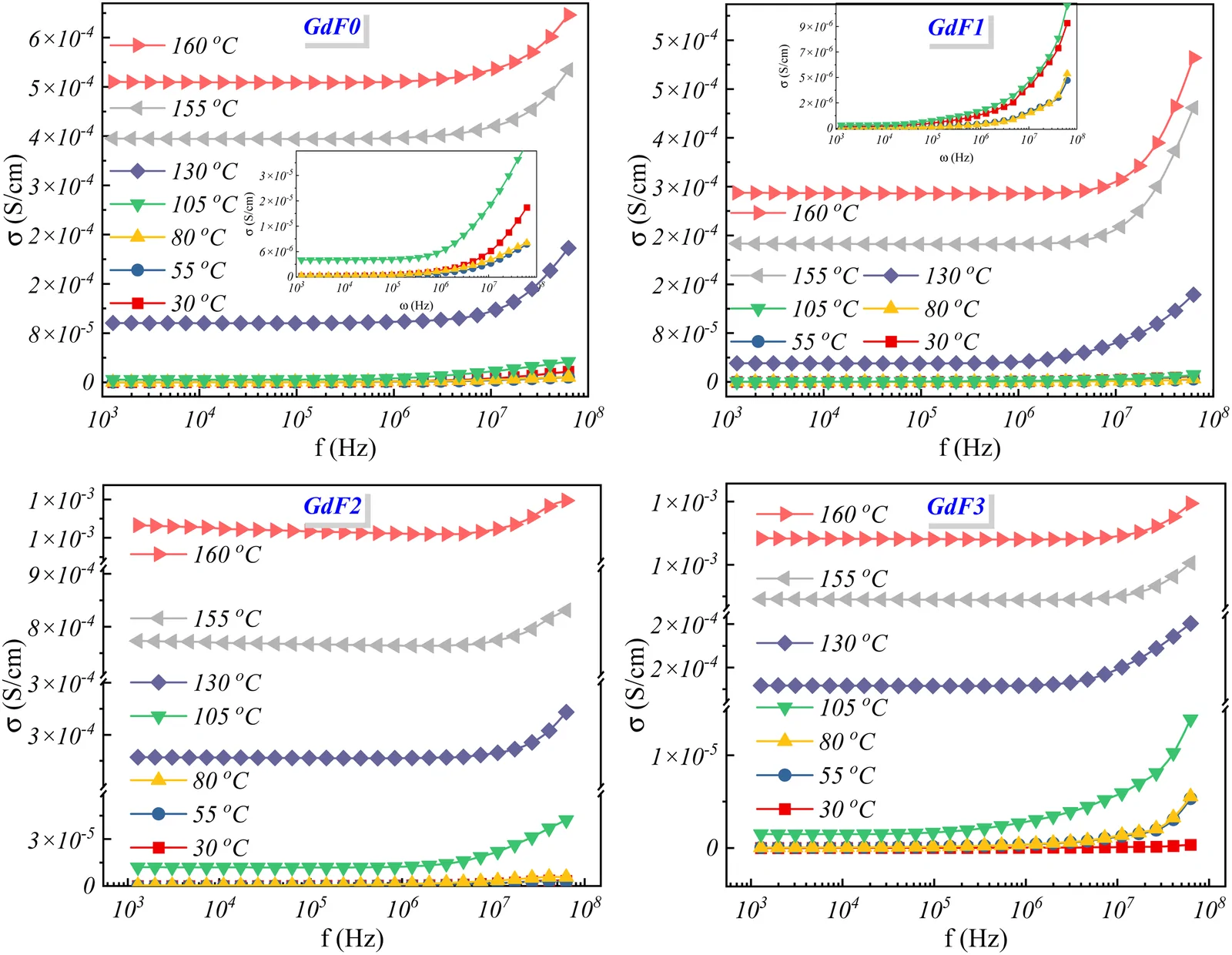
Dependence of the electrical conductivity (*σ*) of the samples on frequency.

In all samples, the curves show a plateau at lower frequencies corresponding to DC conductivity, followed by a steep increase in conductivity with increasing frequency. Beyond a characteristic frequency, all samples show an increase in *σ*_ac_ with increasing frequency. This phenomenon can be described by the Jonscher's universal power law given in eqn (11).^62^

11 *σ*(*ω*) = *σ*_dc_ + *Aω*^*s*^

Here, *σ*_dc_ is the direct current contribution, and *Aω*^*s*^ is the frequency-dependent AC contribution, with *A* being a temperature-dependent constant and s being a constant between 0 and 1.^63^ This contribution is frequency-dependent due to short-range transport of charge carriers, which is attributed to polaron hopping and defect-mediated conduction. In the present work, the Gd-doped samples in particular exhibit a dominant *σ*_dc_ term in most of the measured frequency range, with the *σ*_ac_ term present only to a small extent. This is associated with the increased electron scattering and charge localization in the Gd-doped samples, which is believed to diminish the AC component.^64^ At lower frequencies, the conductivity is almost constant and equal to *σ*_dc_. This is associated with the restricted mobility of charge carriers, as grain boundaries act as barriers that limit ion hopping. In this range, the charge carriers do not have sufficient energy to overcome the potential barriers associated with the hopping between the Fe^3+^ and Fe^2+^ ions in the vicinity of the grain boundary. In this case, it is anticipated that the transport of charge carriers will be governed by the hopping of electrons between the Fe^3+^ and Fe^2+^ ions located at the octahedral sites.^65^ In this region, long-range charge transport predominantly involves electron hopping between Fe^3+^ and Fe^2+^ ions. These ions are located on the octahedral sites in the inverse spinel structure of Fe_3_O_4_. The conductivity exhibits dispersion at higher frequencies, which can be explained by Koop's theory. According to Koop's phenomenological model, the higher *σ*_ac_ at higher frequencies indicates that the material contains more active conducting grains than at lower frequencies.^66^ AC conduction in the literature is explained by small-polaron hopping, defect-induced charge-carrier localization, and grain-boundary interactions, all of which are consistent with our current analysis. The increase in *σ*_dc_ with heating indicates hopping conduction driven by thermal activation and is consistent with the Arrhenius equation. The increase in temperature enhances charge-carrier tunneling, thereby increasing the AC and DC conductivities.

When Gd-doped samples of Fe_3_O_4_ are considered, the AC conductivity shows the same frequency dependence that is also seen in the undoped samples of Fe_3_O_4_. Although Gd incorporation can increase local carrier scattering and localization, the overall conductivity increases, as indicated by the reduced activation energy. The enhanced conductivity is therefore attributed to modification of the charge-transport pathways and Fe^2+^/Fe^3+^ hopping rather than to a simple increase in free-carrier concentration.^61^ These defect states and oxygen vacancies provide hopping sites for charge carriers, thus increasing conductivity. These effects, together with the coexistence of Fe^2+^/Fe^3+^ ions and grain-boundary conduction, can facilitate thermally activated hopping of charge carriers. The observed conductivity therefore supports a defect- and microstructure-influenced hopping mechanism, without requiring a specific concentration of oxygen vacancies. Activation energy (*E*_a_) associated with the charge-transport process was calculated from the Arrhenius analysis of the temperature-dependent electrical conductivity (*σ*) behavior (Fig. S4). The conductivity was analyzed using the Arrhenius relation.

12 *σ* = *σ*_0_ exp(−*E*_a_/*k*_B_*T*)

where *σ*_0_ is a constant, *k*_B_ is the Boltzmann constant, and *T* is the temperature. Thus, *E*_a_ was obtained from the slope of ln(*σ*) *vs.* 1000/*T*. The obtained activation energies are 0.1413 eV, 0.1125 eV, 0.1118 eV, and 0.0864 eV for GdF0, GdF1, GdF2, and GdF3, respectively. The results indicate a decreasing trend in *E*_a_ as Gd doping increases. The results reveal a decreasing trend of *E*_a_ with Gd doping. This reduction indicates that Gd incorporation enables thermally activated charge transport by lowering the energy barrier for carrier hopping. At higher Gd concentrations, the substantially lower activation energy suggests enhanced carrier mobility/hopping probability and modified Fe^2+^/Fe^3+^-mediated charge transport.

#### 3.7.1. Frequency exponent

The frequency exponent, *s*, which is derived from the universal power law, provides valuable insight into the conduction mechanism of the ferrite system in question. The compositional dependence of the frequency exponent *s* is shown in Fig. 9. In the samples, the dominant trend is that the frequency exponent s increases with increasing temperature. This is characteristic behavior of the non-overlapping small-polaron tunneling (NSPT) conduction mechanism. In the NSPT mechanism, charge carriers can jump between two localized states separated by a potential barrier. An increase in temperature provides charge carriers with more potential energy, thereby reducing the barrier and increasing the probability of jumping between states. The frequency exponent of the NSPT mechanism is defined by eqn (13).^67^13
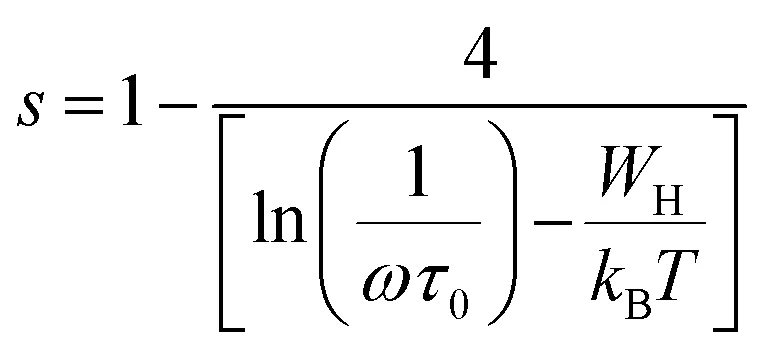
where *W*_H_ is the polaron hopping energy, and *τ*_o_ is a characteristic relaxation time associated with the polaron tunneling process. These parameters are intrinsic to the NSPT model and were not measured in the present study.

**Fig. 9:**
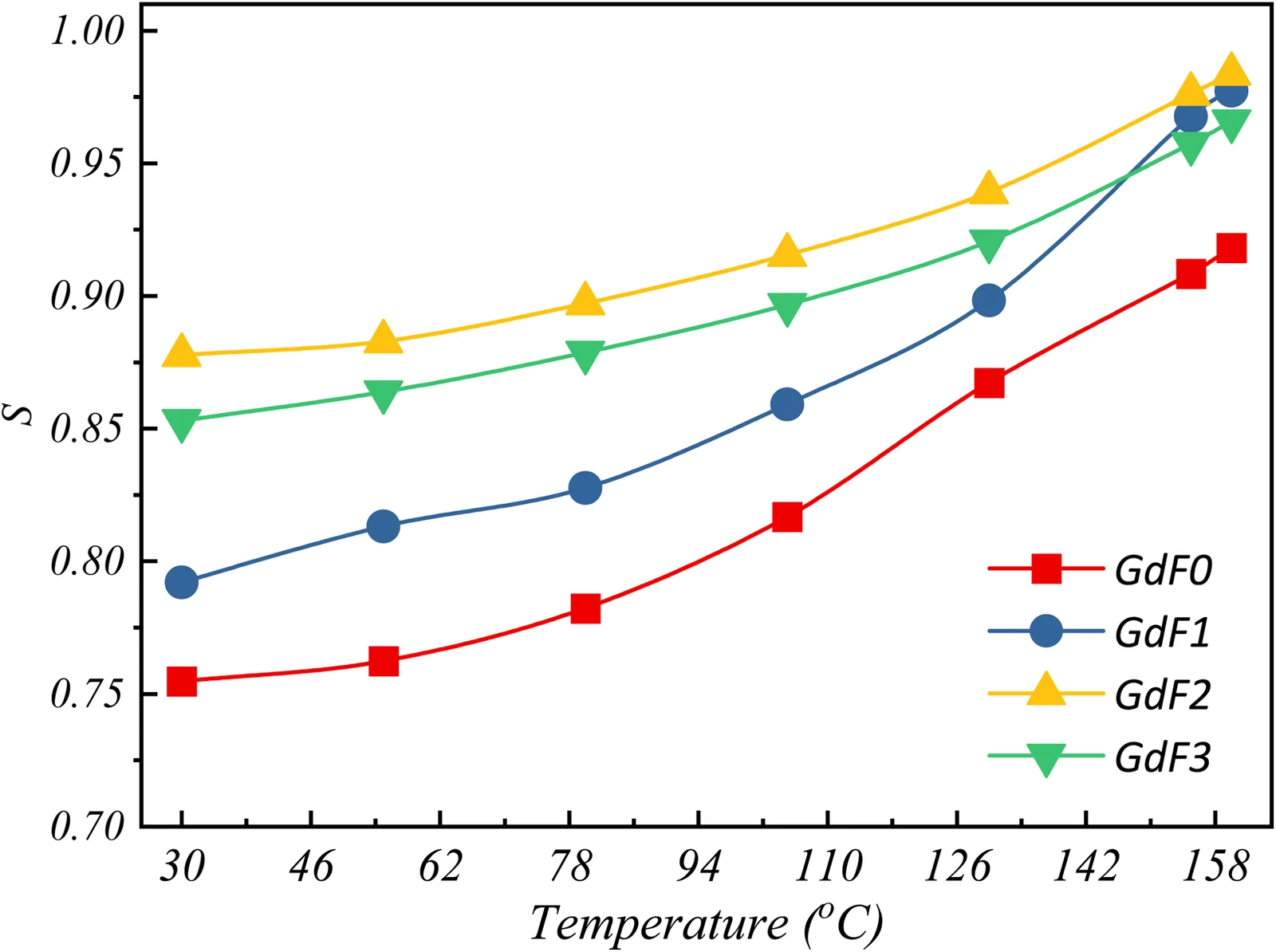
Plots of the frequency exponent ‘*s*’ *versus* temperature.

From Fig. 9, an increasing trend of ‘*s*’ with temperature for all compositions indicates that the conduction mechanism remains essentially the same across the series. Incorporating Gd^3+^ ions into the Fe_3_O_4_ lattice causes lattice distortion and creates defect states and vacancies that provide hopping conduction. These defects lower the effective hopping barrier and improve the thermally activated transport of charge carriers. In addition, the increase in ‘*s*’ at high temperatures can be attributed to increased hopping of electrons and Fe^2+^ and Fe^3+^ ions, decreased grain boundary resistance, and increased participation of defect states. The presence of grain boundaries and structural disorder in the nanocrystalline ferrite system further promotes hopping by providing trapping and release sites for charge carriers. Similar conduction behavior in the Fe_3_O_4_ nanocrystal has also been reported by A. Radon *et al.* at temperatures above room temperature.^68^

### 3.8. VSM analysis

The magnetic characteristics of the synthesized Gd-doped magnetite nanocrystals were studied using vibrating sample magnetometry and are shown in Fig. 10. The hysteresis curves show narrow loops with lower coercivity and remanence, indicating soft magnetic behavior. The obtained magnetic parameters are summarized in Table 1. The lower coercivity reflects relatively easy magnetization reversal and weak magnetic anisotropy, which are characteristic of soft magnetic nanoferrites. The magnetic behavior of ferrites can be described using Néel's two-sublattices model. In this model, the distribution of cations in the tetrahedral (A) and octahedral (B) lattices governs the magnetic behavior, and the A–B superexchange interactions are dominant over the A–A and B–B interactions. The replacement of Fe ions with Gd ions affects these A–B interactions, and as a consequence, the overall magnetization of the ferrite is affected.^69^

**Fig. 10:**
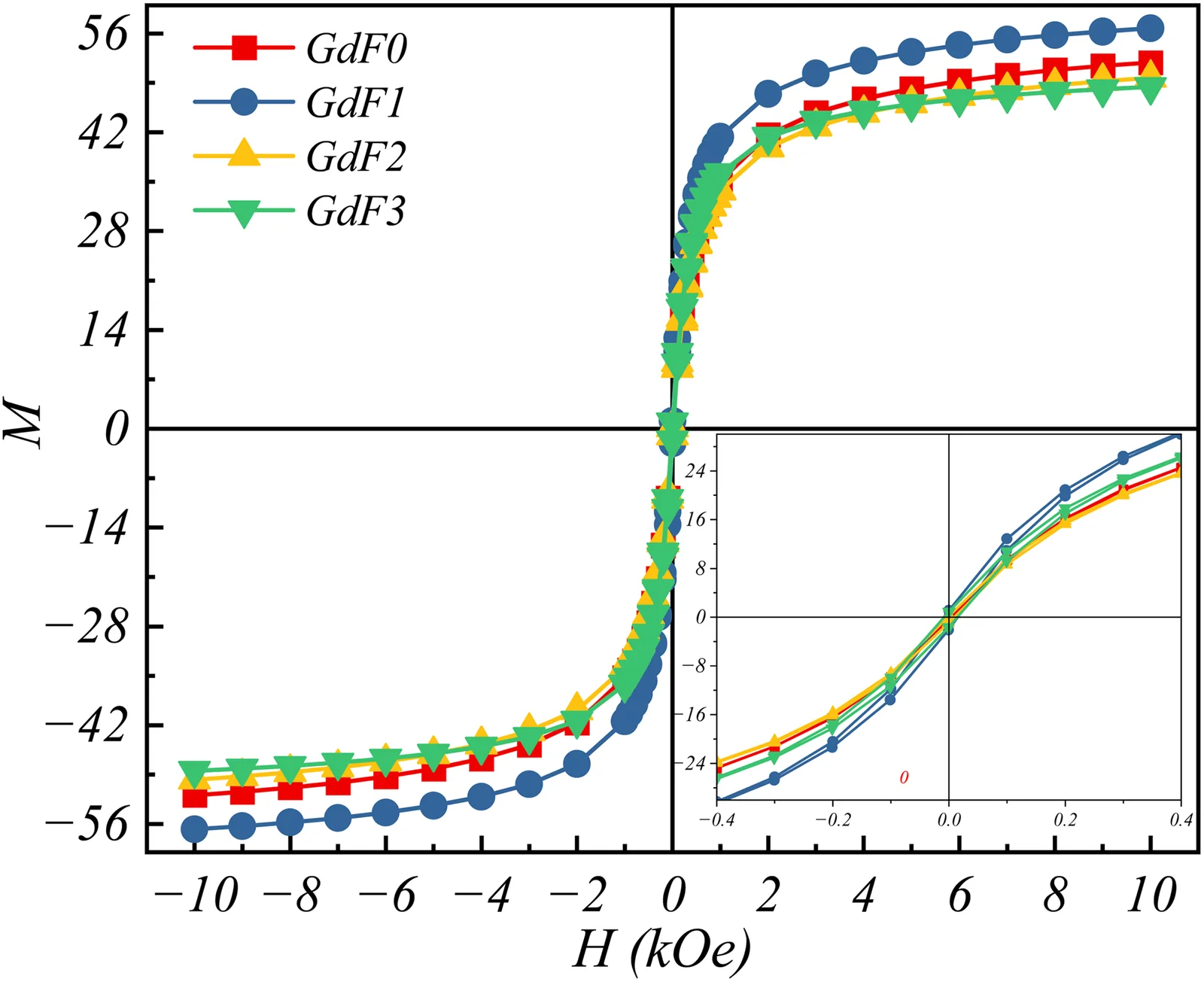
M–H hysteresis loops of the samples.

**Table 1:** Various magnetic parameters obtained from the M–H hysteresis loops

Sample	Saturation magnetisation *M*_s_ (emu g^−1^)	Remanence *M*_r_ (emu g^−1^)	Coercivity *H*_c_ (kOe)	Squareness ratio *M*_r_/*M*_s_
GdF0	50.0	8.5	0.090	0.17
GdF1	55.0	9.8	0.110	0.18
GdF2	46.0	7.9	0.085	0.16
GdF3	48.0	7.2	0.080	0.16

The saturation magnetization of synthesized Fe_3_O_3_ nanoparticles was observed to be similar to that reported in previous studies.^70,71^ All the samples exhibited lower remanence and coercivity, consistent with soft ferrimagnetic behavior. The saturation magnetization shows a non-monotonic variation with Gd concentration. *M*_s_ initially increases from 50.0 emu g^−1^ for GdF0 to 55.0 emu g^−1^ for GdF1, followed by a decrease to 46.0 emu g^−1^ for GdF2 and a slight increase to 48.0 emu g^−1^ for GdF3. The initial increase may be associated with changes in cation distribution and magnetic exchange interactions at lower Gd concentrations, whereas the subsequent decrease may be related to lattice distortion, surface spin disorder, and weakened Fe–O–Fe exchange interactions at higher Gd concentrations. Thus, Gd incorporation modifies rather than decreases the magnetization of the Fe_3_O_4_ nanocrystals.^30^ It has been observed that the *M*_s_ of doped samples (GdF2 and GdF3) is lower than that of bare Fe_3_O_4_. This is attributed to the lack of surface ion coordination, which creates spin disorder or surface spin canting.^72–74^ It has been suggested that the decrease in *M*_s_ of doped samples compared to bare samples is due to the antiferromagnetic coupling of dopant cations to the octahedral site cations of the Fe_3_O_4_ lattice.^34^ Previous studies also reported a decrease in saturation *M*_s_ when Gd was substituted with Gd-doped magnetite nanoparticles.^75,76^

In addition to cation substitution effects, several microstructural factors influence the magnetic behavior. The nanoscale particle size and the high grain-boundary fraction introduce surface spin disorder and canting of magnetic moments, thereby reducing the effective magnetization by weakening long-range magnetic order within the nanoparticles. Moreover, structural defects, lattice strain, and defect-related local environments associated with Gd substitution may modify exchange interactions and contribute to variations in magnetic parameters. Accordingly, all samples exhibit soft ferrimagnetic behavior, with lower remanence and coercivity.^51^ Coercivity is a measure of the magnetic field necessary to demagnetize a ferromagnetic substance. Lower coercivity values indicate that the samples exhibit soft magnetism and support the presence of reduced magnetocrystalline anisotropy due to spin coupling between Fe^3+^/Fe^2+^ and Gd^3+^.^77^ This promotes domain wall motion, and a lower coercivity indicates that magnetization reversal occurs at a small magnetic field, which is typical of soft ferrite materials. The remanent magnetization (*M*_r_) is similarly lower, resulting in a lower squareness ratio (*M*_r_/*M*_s_), indicating the presence of a large number of magnetic particles in the domain. A squareness ratio of *M*_r_/*M*_s_ < 0.5 indicates the presence of multidomain structures.^78^ The results indicate that a small percentage of Gd substitution is sufficient to improve magnetism slightly, whereas higher percentages yield a magnetically decoupled Fe_3_O_4_ lattice with reduced magnetism.

### 3.9. Perspective: energy-related device applications

The structural, electrical, and magnetic studies indicate that the synthesized Gd-doped magnetite nanocrystals exhibit several features beneficial for the development of energy-related devices. The nanocrystalline structure provides a significant fraction of grain boundaries and interfaces, which can contribute to interfacial polarization. Based on the Maxwell–Wagner polarization theory, under an alternating electric field, these grain boundaries, as a result of charge accumulation, increase the effective dielectric permittivity. Improved polarization is a key factor in dielectric capacitors and other energy-storage devices, as it can considerably enhance their storage capability (Scheme 2).

**Scheme 2:**
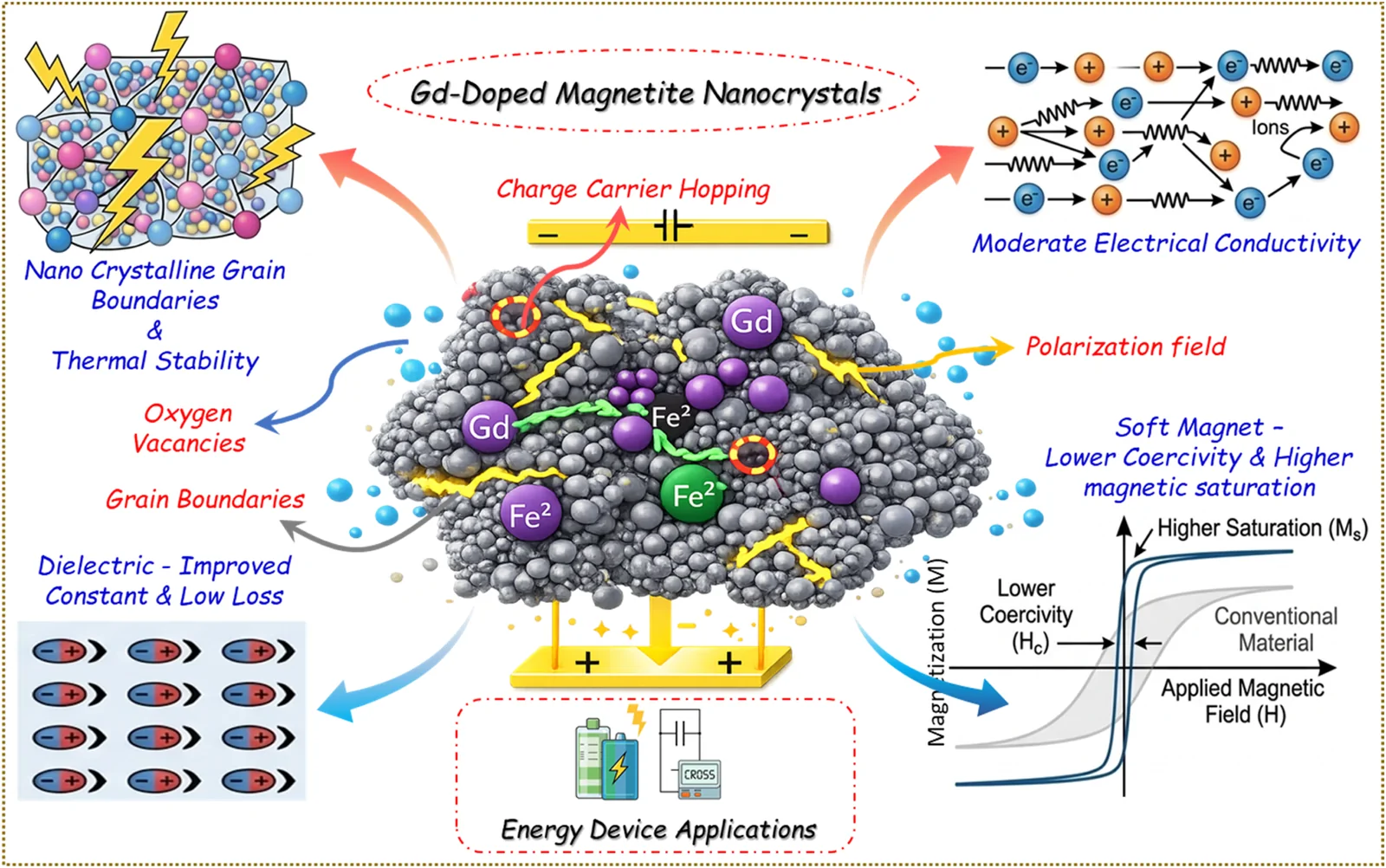
Wide range of applications of Gd-doped Fe_3_O_4_ nanocrystals.

The analysis of electrical conductivity confirms that charge transport occurs *via* thermally activated electron hopping between Fe^2+^ and Fe^3+^ ions at octahedral B sites. This conduction mechanism embodies a balance between electrical and dielectric conductivity. Furthermore, Gd^3+^ substitution introduces noticeable lattice distortion and structural disorder, which, together with grain-boundary heterogeneity, can promote dipolar and interfacial polarization, both of which are key to improving charge mobility and dielectric polarization, providing an added advantage in the use of this material for capacitors, sensors, and other electronic devices. The inherent thermal stability and the soft magnetic characteristics of these materials further enhance their suitability for use in real devices. Good thermal stability ensures dependable operation as conditions change, while soft magnetic behavior with lower coercivity helps minimize losses and interference. Thus, the observed combination of nanostructure, dielectric polarization, charge transport, thermal stability, and magnetic characteristics provides a basis for exploring these materials in future dielectric, energy-related, and multifunctional electronic devices.

## 4. Conclusion

Gd-doped magnetite nanocrystals were successfully synthesized, and their dielectric and magnetic properties were systematically investigated. The XRD analysis confirms the retention of the Fe_3_O_4_ cubic spinel structure without any secondary phases even after the addition of Gd. With increasing Gd content, the crystallite size increased from 7.24 to 14.81 nm, whereas the microstrain and dislocation density decreased from 13.49 × 10^−3^ to 6.47 × 10^−3^ and 19.08 × 10^15^ to 4.56 × 10^15^ m^−2^, respectively, indicating reduced structural disorder. Gd-doped magnetite structures also exhibit enhanced electron hopping due to the coexistence of Fe oxidation states. The dielectric results revealed a frequency- and temperature-dependent response, governed mainly by interfacial polarization and thermally activated charge-carrier hopping. The dielectric loss decreased with increasing frequency and Gd concentration, indicating reduced dielectric dissipation. The AC conductivity and frequency-exponent analysis further supported a thermally activated hopping mechanism primarily associated with Fe^2+^/Fe^3+^ charge transfer. The magnetic measurements revealed soft ferrimagnetic behavior with lower coercivity (80–110 Oe) and finite remanent magnetization (7.2–9.8 emu g^−1^). The combined dielectric, electrical, thermal, and magnetic characteristics indicate that Gd-doped Fe_3_O_4_ nanocrystals may have potential for future dielectric, energy-related, and electronic applications.

A limitation of the present study is that independent synthesis replicates and repeated measurements were not performed; therefore, sample-to-sample reproducibility and statistical uncertainty could not be independently assessed. Future studies involving independent batch synthesis and repeated measurements, together with direct device-level evaluation of energy-storage performance, are needed to further establish the reproducibility and practical applicability of these materials.

## Conflicts of interest

There are no conflicts to declare.

## Supplementary Material

RA-OLF-D6RA06350K-s001

## Data Availability

The data are available upon request. Supplementary information (SI) is available. See DOI: https://doi.org/10.1039/d6ra06350k.

## References

[cit1] Saraçouglu M., Mansoor M., Bakirdöven U., Arpali H., Gezici U. O., Timur S. (2023). Challenging the frontiers of superparamagnetism through strain engineering: DFT investigation and co-precipitation synthesis of large aggregated Fe_3_O_4_ (magnetite) powder. J. Alloys Compd..

[cit2] Imran M., Haider A., Shahzadi I., Moeen S., Ul-Hamid A., Nabgan W., Shahzadi A., Alshahrani T., Ikram M. (2023). others, Polyvinylpyrrolidone and chitosan-coated magnetite (Fe_3_O_4_) nanoparticles for catalytic and antimicrobial activity with molecular docking analysis. J. Environ. Chem. Eng..

[cit3] Rahman M. T., Vargas M., Ramana C. V. (2014). Structural characteristics, electrical conduction and dielectric properties of gadolinium substituted cobalt [1]. J. Alloys Compd..

[cit4] Thang P. D., Rijnders G., Blank D. H. A. (2005). Spinel cobalt ferrite by complexometric synthesis. J. Magn. Magn. Mater..

[cit5] Bharathi K. K., Ramana C. V. (2011). Improved electrical and dielectric properties of La-doped Co ferrite. J. Mater. Res..

[cit6] Bharathi K. K., Noor-A-Alam M., Vemuri R. S., Ramana C. V. (2012). Correlation between microstructure, electrical and optical properties of nanocrystalline NiFe 1.925 Dy 0.075 O 4 thin films. RSC Adv..

[cit7] Gu Z., Xiang X., Fan G., Li F. (2008). Facile Synthesis and Characterization of Cobalt Ferrite Nanocrystals via a Simple Reduction–Oxidation Route. J. Phys. Chem. C.

[cit8] Gul I. H., Maqsood A. (2008). Structural, magnetic and electrical properties of cobalt ferrites prepared by the sol–gel route. J. Alloys Compd..

[cit9] Kumari S., Dalal J., Kumar A., Ohlan A. (2022). Microwave Absorption Performance of Core–Shell rGO/Ni0.5Co0.5Fe_2_O_4_@PEDOT Composite: An Effective Approach to Reduce Electromagnetic Wave Pollution. Adv. Eng. Mater..

[cit10] Sundararajan M., Sukumar M., Dash C. S., Sutha A., Suresh S., Ubaidullah M., Al-Enizi A. M., Raza M. K., Kumar D. (2022). A comparative study on NiFe_2_O_4_ and ZnFe_2_O_4_ spinel nanoparticles: Structural, surface chemistry, optical, morphology and magnetic studies. Phys. B Condens. Matter.

[cit11] Singh V. (2025). An overview on recent trends of spinel ferrites (MFe 2 O 4: M = Fe^2+^, Co^2+^, Mn^2+^, Ni^2+^, Zn^2+^) synthesis and catalytic applications. Int. J. New Chem..

[cit12] Dalal J., Lather S., Gupta A., Tripathi R., Maan A. S. (2019). Reduced Graphene Oxide Functionalized Strontium Ferrite in Poly ( 3 , 4-ethylenedioxythiophene ) Conducting Network : A High-Performance EMI Shielding Material. Adv. Mater. Technol..

[cit13] Kadian N., Kumari R., Panchal A., Dalal J., Padalia D. (2023). Structural and Optical Properties of Gadolinium Doped-Magnetite Nano-crystal for Photocatalytic Application. J. Alloys Compd..

[cit14] Dalal J., Malik S., Dahiya S., Punia R., Singh K., Maan A. S., Dhawan S. K., Ohlan A. (2021). One pot synthesis and electromagnetic interference shielding behavior of reduced graphene oxide nanocomposites decorated with Ni0.5Co0.5Fe_2_O_4_ nanoparticles. J. Alloys Compd..

[cit15] Almulaiky Y. Q., Al-Harbi S. A. (2025). Innovative immobilization of $β$-glucosidase on amidoximated acrylic fabric integrated with magnetite Fe3O4 nanoparticles. Int. J. Biol. Macromol..

[cit16] Kohale M., Inamdar H., Kokate K., Ingale R., Joshi J., Singh D., Katti A., Polshettiwar S., Aher R., Kulkarni S. (2026). Engineering magnetite (Fe3O4) nanoparticles: Controlled synthesis, surface functionalization, and multidisciplinary technological applications: A Review. Prog. Cryst. Growth Charact. Mater..

[cit17] Begum S. K., Shabnam D., Haque N., Alam M. J., Ferdous J., Nur U. J. B., Fatema K., Shabiha R. J., Clarke R. J., Chowdhury P. (2025). others, Green synthesis of magnetite (Fe3O4) and hematite (Fe2O3) nanoparticles using *Moringa oleifera* and Psidium guajava leaf extracts for sustainable applications. Sci. Rep..

[cit18] Kumari S., Sharma E., Verma J., Dalal J., Kumar A. (2023). Structural and photoluminescence properties of Dy-doped nanocrystalline ZrO2 for optoelectronics application. Ceram. Int..

[cit19] Paulsen J. A., Ring A. P., Lo C. C. H., Snyder J. E., Jiles D. C. (2005). Manganese-substituted cobalt ferrite magnetostrictive materials for magnetic stress sensor applications. J. Appl. Phys..

[cit20] Vereda F., de Vicente J., Hidalgo-Álvarez R. (2008). Synthesis of Ni ferrite and Co ferrite rodlike particles by superposition of a constant magnetic field. J. Mater. Res..

[cit21] Yunasfi A. M., Mashadi, Winatapura D. S., Wisnu A. A. (2021). Magnetic and microwave absorption properties of La^3+^-substituted manganese ferrites synthesized via solid-state reaction method. Appl. Phys. A.

[cit22] Gilani Z. A., Warsi M. F., Khan M. A., Shakir I., Shahid M., Anjum M. N. (2015). Impacts of neodymium on structural, spectral and dielectric properties of LiNi0.5Fe2O4 nanocrystalline ferrites fabricated via micro-emulsion technique. Phys. E.

[cit23] Käämbre T., Vanags M., Pärna R., Kisand V., Ignatans R., Kleperis J., Šutka A. (2018). Yttrium-doped hematite photoanodes for solar water splitting: Photoelectrochemical and electronic properties. Ceram. Int..

[cit24] Kamakshi T., Sunita Sundari G., Erothu H., Subhakaran Singh R. (2019). Effect Of Nickel Dopant On Structural, Morphological And Optical Characteristics Of Fe3o4 Nanoparticles. Rasayan J. Chem..

[cit25] Aghazadeh M., Ganjali M. R. (2018). Samarium-doped Fe_3_O_4_ nanoparticles with improved magnetic and supercapacitive performance: a novel preparation strategy and characterization. J. Mater. Sci..

[cit26] Andrade Neto N. F., Nascimento L. E., Correa M., Bohn F., Bomio M. R. D., Motta F. V. (2020). Characterization and photocatalytic application of Ce^4+^, Co^2+^, Mn^2+^ and Ni^2+^ doped Fe_3_O_4_ magnetic nanoparticles obtained by the co-precipitation method, Mater. Chem. Phys..

[cit27] Zeng M., Thummavichai K., Chen W., Liu G., Li Z., Chen X., Feng C., Li Y., Wang N., Zhu Y. (2021). Study on the mechanism of tunable ferromagnetic composites with different rare earth ions. RSC Adv..

[cit28] Padalia D., Kumar U., Bhandari P., Dalal J., Ranakoti L., Singh T. (2024). Tuning the structural, optical, and dielectric properties of europium-doped barium titanate ceramics. J. Mater. Sci.:Mater. Electron..

[cit29] Kumari R., Kadyan N., Korpal S., Kumar S., Dalal J., Thanasekaran P. (2025). others, Tailoring Dielectric and Electrical Properties of Gd Doped BaTiO3 Ceramics for Energy Device Applications. J. Mater. Res. Technol..

[cit30] Joshi S., Kumar M., Pandey H., Singh M., Pal P. (2018). Structural, magnetic and dielectric properties of Gd^3+^ substituted NiFe2O4 nanoparticles. J. Alloys Compd..

[cit31] Kumari R., Kumari S., Sharma E., Kumari S., Dalal J., Padalia D. (2023). Structural and optical properties of gadolinium doped barium titanate nano-crystal for multifunctional
nanodevices. Opt. Mater..

[cit32] Somvanshi S. B., Khedkar M. V., Kharat P. B., Jadhav K. M. (2020). Influential diamagnetic magnesium (Mg^2+^) ion substitution in nano-spinel zinc ferrite (ZnFe2O4): Thermal, structural, spectral, optical and physisorption analysis. Ceram. Int..

[cit33] Pawar R. A., Patange S. M., Shitre A. R., Gore S. K., Jadhav S. S., Shirsath S. E. (2018). Crystal chemistry and single-phase synthesis of Gd^3+^ substituted Co–Zn ferrite nanoparticles for enhanced magnetic properties. RSC Adv..

[cit34] Bavandpour R., Karimi-Maleh H., Asif M., Gupta V. K., Atar N., Abbasghorbani M. (2016). Liquid phase determination of adrenaline uses a voltammetric sensor employing CuFe2O4 nanoparticles and room temperature ionic liquids. J. Mol. Liq..

[cit35] Sun J., Li J., Sun G. (2002). Effects of La2O3 and Gd2O3 on some properties of Ni–Zn ferrite. J. Magn. Magn. Mater..

[cit36] Hazarika K. P., Fopase R., Pandey L. M., Borah J. P. (2022). Influence of Gd-doping on structural, magnetic, and self-heating properties of Fe3O4 nanoparticles towards magnetic hyperthermia applications. Phys. B Condens. Matter.

[cit37] Faramawy A. M., Elsayed H., Elsayed H. M., Sattar A. A., Getahun Y. W., El-Gendy A. A., Kahil H. (2024). Unveiling the effect of Gd^3+^ doping on enriching the structural, magnetic, optical, and dielectric properties of biocompatible hematite nanoparticles. J. Alloys Compd..

[cit38] Badr M. H., Fadl N. A., EL-Hamalawy A. A., Abouhaswa A. S. (2024). Influence of annealing temperature on microstructure, magnetic and electrical properties of Ce doped cobalt ferrite nanoparticles. Ceram. Int..

[cit39] Vinod G., Rajashekhar K., Sandeep Y., Laxman Naik J. (2022). Influence of RE-Gd^3+^ ion substitution on structure, morphology, optical, and magnetic analysis of Cu-Cd based nano ferrites synthesized by low-temperature citrate sol–gel auto combustion method. J. Magn. Magn. Mater..

[cit40] Apostolova I., Apostolov A., Wesselinowa J. (2023). Magnetic Properties of Gd-Doped Fe_3_O_4_ Nanoparticles. Appl. Sci..

[cit41] Munimasthani S., Sarathkumar S., Udaychandran Thampy U. S., Ravikumar R. (2020). Structural and luminescence studies of Dy^3+^-activated cadmium calcium pyrophosphate. Appl. Phys. A.

[cit42] Debnath S., Deb K., Saha B., Das R. (2019). X-ray diffraction analysis for the determination of elastic properties of zinc-doped manganese spinel ferrite nanocrystals (Mn0.75Zn0.25Fe_2_O4), along with the determination of ionic radii, bond lengths, and hopping lengths. J. Phys. Chem. Solids.

[cit43] Dalal J., Lather S., Gupta A., Dahiya S., Maan A. S., Singh K., Dhawan S. K., Ohlan A. (2018). EMI shielding properties of laminated graphene and PbTiO3 reinforced poly (3, 4-ethylenedioxythiophene) nanocomposites. Compos. Sci. Technol..

[cit44] Lesiak B., Rangam N., Jiricek P., Gordeev I., Tóth J., Kövér L., Mohai M., Borowicz P. (2019). Surface Study of Fe_3_O_4_ Nanoparticles Functionalized With Biocompatible Adsorbed Molecules. Front. Chem..

[cit45] Fumis D. B., Silveira M. L. D., Gaglieri C., Ferreira L. T., Marques R. F. C., Magdalena A. G. (2022). The Effect of EDTA Functionalization on Fe3O4 Thermal Behavior. Mater. Res..

[cit46] Murtaza A., Song X., Noreen S., Zhang W., Haoyi W., Ghani A., Kabir F., Zuo W.-L., Li K., Saeed A., Yang S. (2025). Oxygen vacancies mediated ferromagnetism with enhanced optical and dielectric properties in Gd^3+^ and Co^2+^/Mn^2+^ co-doped ZnO nanocrystals. Results Phys..

[cit47] Rao T. G. V. M., Rupesh Kumar A., Hanumantha Rao B., Veeraiah N., Rami Reddy M. (2012). Optical absorption, ESR, FT-IR spectral studies of iron ions in lead oxyfluoro silicate glasses. J. Mol. Struct..

[cit48] Mohamed W. S., Tozri A., Abdelbaky M. S. M., Garcia-Granda S., Almutairi T. S., Alzaid M., Abu-Dief A. M. (2024). Effect of heavy Dy^3+^ doping on the magnetic, structural, morphological, and optical characteristics of CuDyxFe_2-x_O_4_ nanoparticles. Ceram. Int..

[cit49] Coduri M., Masala P., Del Bianco L., Spizzo F., Ceresoli D., Castellano C., Cappelli S., Oliva C., Checchia S., Allieta M. (2020). others, Local structure and magnetism of Fe2O3 maghemite nanocrystals: The role of crystal dimension. Nanomaterials.

[cit50] Joshi S., Kumar M. (2016). Influence of Co^2+^ substitution on cation distribution and on different properties of NiFe 2 O 4 nanoparticles. J. Supercond. Nov. Magn..

[cit51] Alotaibi I., Alshammari M. S., Algessair S., Madkhali N., All N. A., Hjiri M., Alrub S. A., El Mir L., Lemine O. M. (2022). Synthesis, characterization and heating efficiency of Gd-doped maghemite (γ-Fe2O3) nanoparticles for hyperthermia application. Phys. B Condens. Matter.

[cit52] Lather S., Dalal J., Gupta A., Singh S., Singh D. P., Dahiya S. (2019). PbTiO3–Ni0.5Co0.5Fe_2_O_4_ multiferroic nanocomposites: Impact of ball-milling on dielectric, magnetic and ferroelectric properties. Ceram. Int..

[cit53] Lather S., Dalal J., Gupta A., Singh S., Singh D. P., Dahiya S., Maan A. S., Tripathi R., Ohlan A. (2019). PbTiO_3_–Ni_0.5_Co_0.5_Fe_2_O_4_ multiferroic nanocomposites: Impact of ball-milling on dielectric, magnetic and ferroelectric properties. Ceram. Int..

[cit54] Ahmed M. A., Okasha N., Kershi R. M. (2009). Extraordinary role of rare-earth elements on the transport properties of barium W-type hexaferrite. Mater. Chem. Phys..

[cit55] Bharathi K. K., Markandeyulu G., V Ramana C. (2011). Structural, magnetic, electrical, and magnetoelectric properties of Sm-and Ho-substituted nickel ferrites. J. Phys. Chem. C.

[cit56] Koops C. G. (1951). On the Dispersion of Resistivity and Dielectric Constant of Some Semiconductors at Audio frequencies. Phys. Rev..

[cit57] Iwauchi K. (1971). Dielectric properties of fine particles of Fe3O4 and some ferrites. Jpn. J. Appl. Phys..

[cit58] Ramana Reddy A. V., Ranga Mohan G., Ravinder D., Boyanov B. S. (1999). High-frequency dielectric behaviour of polycrystalline zinc substituted cobalt ferrites. J. Mater. Sci..

[cit59] Verma A., Thakur O. P., Prakash C., Goel T. C., Mendiratta R. G. (2005). Temperature dependence of electrical properties of nickel–zinc ferrites processed by the citrate precursor technique. Mater. Sci. Eng., B.

[cit60] Zhang Y., Ma T., Wang X., Yuan Z., Zhang Q. (2011). Two dielectric relaxation mechanisms observed in lanthanum doped barium strontium titanate glass ceramics. J. Appl. Phys..

[cit61] Rahman M. T., Vargas M., Ramana C. V. (2014). Structural characteristics, electrical conduction and dielectric properties of gadolinium substituted cobalt ferrite. J. Alloys Compd..

[cit62] Roy A., Ponnam A., Varade V., V Honnavar G., Menon R. (2023). Evidence of Lampert Triangle and Jonscher's Double Power Law in Doped Poly(3,4-ethylenedioxythiophene) Devices. J. Phys. Chem. C.

[cit63] Dhahri R., Benamara M., Bouzidi S., Ben Moussa S., Alzahrani A. Y. A., Nassar K. I., Zahmouli N., Elkenany E. B., Al-Syadi A. M. (2025). Effect of Gd doping on the microstructure and electrical characteristics of Maghemite (γ-Fe_2_O_3_) ceramics. J. Sol-Gel Sci. Technol..

[cit64] Srour A., Bitar Z., Badreddine K., Awad R. (2023). Physical properties and dielectric response of (Gd, Pr)-dual doped samarium iron garnet. Ceram. Int..

[cit65] Anitha Reddy R., Venkateswara Rao A., Rajesh Babu B., Rama Rao K., Raghavendra V. (2023). Structural, magnetic and antibacterial studies of gadolinium doped cobalt ferrite nanoparticles synthesized at low temperature. Adv. Nat. Sci.:Nanosci. Nanotechnol..

[cit66] Melagiriyappa E., Jayanna H. S., Chougule B. K. (2008). Dielectric behavior and ac electrical conductivity study of Sm^3+^ substituted Mg–Zn ferrites. Mater. Chem. Phys..

[cit67] Kumar S., Kumari S., Kumar V., Dalal J., Kumar A., Ohlan A. (2022). Electrical conductivity and relaxation phenomena in Li_2_O B_2_O_3_ based glass and glass-ceramic: A comprehensive and comparative analysis. J. Phys. Chem. Solids.

[cit68] Radoń A., Łukowiec D., Kremzer M., Mikuła J., Włodarczyk P. (2018). Electrical Conduction Mechanism and Dielectric Properties of Spherical Shaped Fe3O4 Nanoparticles Synthesized by Co-Precipitation Method. Materials.

[cit69] Kakde A. S., Belekar R. M., Wakde G. C., Borikar M. A., Rewatkar K. G., Shingade B. A. (2021). Evidence of magnetic dilution due to unusual occupancy of zinc on B-site in NiFe2O4 spinel nano-ferrite. J. Solid State Chem..

[cit70] de la Presa P., Luengo Y., Multigner M., Costo R., Morales M. P., Rivero G., Hernando A. (2012). Study of Heating Efficiency as a Function of Concentration, Size, and Applied Field in γ-Fe_2_O_3_ Nanoparticles. J. Phys. Chem. C.

[cit71] Tadic M., Kralj S., Lalatonne Y., Motte L. (2019). Iron oxide nanochains coated with silica: Synthesis, surface effects and magnetic properties. Appl. Surf. Sci..

[cit72] Peddis D., Cannas C., Musinu A., Ardu A., Orrù F., Fiorani D., Laureti S., Rinaldi D., Muscas G., Concas G., Piccaluga G. (2013). Beyond the Effect of Particle Size: Influence of CoFe 2 O 4 Nanoparticle Arrangements on Magnetic Properties. Chem. Mater..

[cit73] Aquino R., Depeyrot J., Sousa M. H., Tourinho F. A., Dubois E., Perzynski R. (2005). Magnetization temperature dependence and freezing of surface spins in magnetic fluids based on ferrite nanoparticles. Phys. Rev. B:Condens. Matter Mater. Phys..

[cit74] Coey J. M. D. (1971). Noncollinear Spin Arrangement in Ultrafine Ferrimagnetic Crystallites. Phys. Rev. Lett..

[cit75] Douglas F. J., MacLaren D. A., Maclean N., Andreu I., Kettles F. J., Tuna F., Berry C. C., Castro M., Murrie M. (2016). Gadolinium-doped magnetite nanoparticles from a single-source precursor. RSC Adv..

[cit76] Thorat N. D., Bohara R. A., Yadav H. M., Tofail S. A. M. (2016). Multi-modal MR imaging and magnetic hyperthermia study of Gd doped Fe 3 O 4 nanoparticles for integrative cancer therapy. RSC Adv..

[cit77] Ghasemi A. (2014). Development of uniaxial magnetocrystalline anisotropy in SrFe_12−*x*_(DyGd)_x_O19 thin film synthesized by incorporation of high coercivity nanoparticles in sol–gel method. J. Magn. Magn. Mater..

[cit78] Sudakar C., Subbanna G. N., Kutty T. R. N. (2003). Wet chemical synthesis of multicomponent hexaferrites by gel-to-crystallite conversion and their magnetic properties. J. Magn. Magn. Mater..

