# Supplementary material for: Dielectric polarization and charge transport in Gd-doped Fe3O4 nanocrystals for energy storage and electronic devices
Source: RSC Adv. 2026 Sep 28;16(45):57828–43. doi: 10.1039/d6ra06350k (PMC13618065; doi:10.1039/d6ra06350k)
Supplement: Supplementary file 1 [file RA-016-D6RA06350K-s001.pdf]

Supplementary Information (SI) for RSC Advances.
This journal is © The Royal Society of Chemistry

Supporting Data

Dielectric Polarization and Charge Transport in Gd-Doped Fe_3O_4 Nanocrystals for Energy Storage and Electronic Devices

Neeraj Kadian[1], Mutaz Salih[3], Renu Kumari[1], Smita Korpal[2], Jasvir Dalal [2,], Ahmed B.M. Ibrahim[3]*

[1] *Department of Physics, Government College for Women, Rohtak-124001, India*

[2] *Department of Physics, Rajdhani College, University of Delhi, Delhi-110015, India*

[3] *Department of Chemistry, College of Science, Imam Mohammad Ibn Saud Islamic University (IMSIU), Riyadh 11623, Saudi Arabia*

1. Experimental Details

1.1. Synthesis

The Gd-doped magnetite ($Gd_xFe_{3-x}O_4$) nanocrystals were synthesized via an alkaline co-precipitation method in an aqueous medium. Analytical grade ferric chloride hexahydrate ($FeCl_3{\cdot}6H_2O$), ferrous chloride tetrahydrate ($FeCl_2{\cdot}4H_2O$) gadolinium chloride hexahydrateand ($GdCl_3{\cdot}6H_2O$) were used as precursor materials and weighed according to the required stoichiometric ratios. Initially, 4.72 g of $FeCl_3{\cdot}6H_2O$ and 1.72 g of $FeCl_2{\cdot}4H_2O$ were dissolved in 80 mL of deionized water to obtain a homogeneous yellow-orange solution. The solution was then heated at 60 °C under continuous magnetic stirring for 80 minutes to ensure complete dissolution and uniform mixing.

Afterward, ammonium hydroxide (NH_4OH) was added dropwise as a precipitating agent until the pH of the solution reached approximately 11, leading to the formation of a black precipitate. To control particle growth and prevent oxidation of Fe_3O_4 nanoparticles, polyethylene glycol (PEG-600) was introduced as a capping agent during the synthesis process. The resulting precipitate was separated using a magnetic field and repeatedly washed with deoxygenated water to remove residual impurities, including ammonium chloride and excess surfactant. Finally, the obtained material was dried in a vacuum oven at 60 °C for 12 hours.

---

* Corresponding author email: jasvirdalal2012@gmail.com (J. Dalal)

A series of four samples with varying gadolinium content ($x = 0.000, 0.015, 0.025$, and 0.050) were prepared following the same procedure. The samples were designated as GdF0, GdF1, GdF2, and GdF3, respectively.

1.2. Characterization Techniques

The synthesized samples were systematically investigated for their structural, thermal, dielectric, electrical, morphological, and magnetic properties using various characterization techniques. The structural characterization of the samples was performed using X-ray diffraction (XRD) with an X'Pert PRO diffractometer (PANalytical). The measurements were performed at a scanning rate of 2°/min over a 2θ range of 20° to 70°. Thermal behavior was analyzed using a TA Instruments system (Model: Q200) at a constant heating rate of 10 °C min^{-1}. The chemical bonding and functional groups were examined using a Fourier transform infrared (FTIR) spectrometer (PerkinElmer, Spectrum Two) in the wavenumber range of 400-4000 cm^{-1} with a resolution of 1 cm^{-1}. X-ray photoelectron spectroscopy (XPS) measurements were performed on a Thermo Scientific Escalab QXi system to examine the elemental composition, oxidation states, and oxygen-vacancy-related defects in the samples. Surface morphology and particle size were analyzed using a JEOL 7610F Plus field emission scanning electron microscope (FE-SEM). Dielectric properties were measured using a Novocontrol Technologies impedance analyzer over a frequency range of 10 Hz to 1 MHz and a temperature range of 30-160 °C. For these measurements, the powdered samples were pelletized using a hydraulic press under a load of 10 tons, producing pellets of approximately 10 mm in diameter and ~2.2 mm thickness. Magnetic properties were evaluated using a vibrating sample magnetometer (VSM, Microsense Model ADE-EV9) to determine key magnetic parameters, including magnetization and coercivity.

2. Results and Discussion

2.1. XRD Analysis

The structural parameters obtained from the Rietveld analysis of XRD data are summarized in Table S1. The χ^2 values obtained at convergence range from 1.24 to 1.39, indicating satisfactory agreement between the experimental and calculated XRD profiles and supporting the reliability of the Rietveld refinement. With increasing Gd concentration from x = 0.000 to 0.050, the lattice parameter increases from 8.3484 to 8.3665 Å, accompanied by an increase in the unit-cell volume from 581.84 to 585.64 Å^3. This systematic expansion of the lattice can be attributed primarily to the larger ionic radius of Gd^{3+} (0.95 Å) compared with Fe^{3+} (0.65 Å). The incorporation of Gd^{3+} into the Fe_3O_4 lattice therefore produces a progressive expansion of the cubic spinel unit cell.

Table S1. *The structural parameters details of $Gd_xFe_{3-x}O_4$ nanoparticles obtained from Rietveld refinement.*

x	Lattice parameter (a, Å)	Volume (V, Å^3)	Crystallite size (D, nm)	Density (ρ$_x$, g/cm^{-3})	Micro-strain ($\times 10^{-3}$)	Dislocation density ($\times 10^{15}$ m^{-2})	Chi-square values at convergence	Grain size from FESEM images (nm)
0.000	8.3484	581.84	7.24	5.286	13.49	19.08	1.39	9.60
0.015	8.3574	583.73	11.18	5.303	8.90	8.0	1.29	12.65
0.025	8.3639	585.09	11.40	5.314	8.45	7.69	1.35	14.15
0.050	8.3665	585.64	14.81	5.367	6.47	4.56	1.24	16.90

The crystallite size increases from 7.24 nm for x = 0.000 to 14.81 nm for x = 0.050. The increase in crystallite size with Gd incorporation suggests that the presence of Gd^{3+} influences the crystallization and growth of the Fe_3O_4 nanocrystals. In contrast, the microstrain decreases systematically from 13.49 $\times 10^{-3}$ to 6.47 $\times 10^{-3}$ as the Gd concentration increases. This inverse relationship between crystallite size and microstrain indicates a progressive reduction in lattice distortion with increasing crystallite size. The X-ray density also increases from 5.286 to 5.367 g cm^{-3} with increasing Gd concentration. This increase can be attributed to the substantially higher atomic mass of Gd relative to Fe, which increases the molecular mass of the composition, while the change in lattice parameter remains relatively small.

Furthermore, the calculated dislocation density decreases markedly from 19.08×10^{15} m^{-2} for x = 0.000 to 4.56×10^{15} m^{-2} for x = 0.050. Since dislocation density is inversely related to the square of crystallite size, the observed decrease is consistent with the increase in crystallite size. The simultaneous reduction in microstrain and dislocation density indicates a decrease in structural defects and lattice distortion as the Gd content increases.

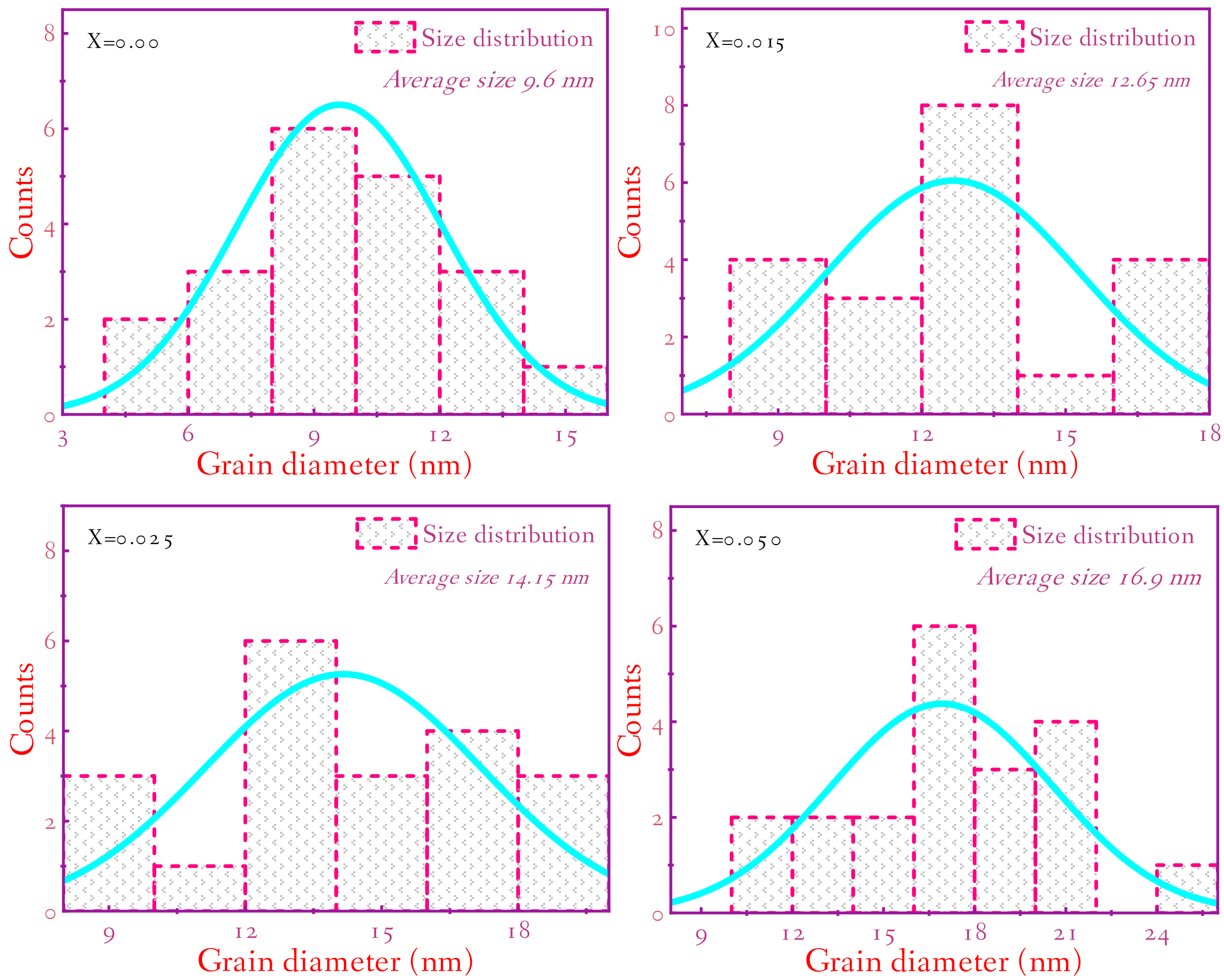

Figure S1: *FESEM Particle/grain size distribution of all samples.*

2.2. Dielectric Loss Analysis

Figure S2 presents the variation of dielectric loss (tanδ) as a function of frequency for all the Fe_3O_4 nanocrystals at different temperatures. For all compositions, tanδ exhibits a noticeable frequency dependence, with relatively higher dielectric loss at lower frequencies, followed by a continuous

decrease as frequency increases. The magnitude of tanδ also increases with increasing temperature, indicating the thermally activated nature of the dielectric-loss mechanism. The relatively higher dielectric loss at lower frequencies can be attributed to the accumulation of charge carriers at grain boundaries and interfaces, resulting in space-charge or Maxwell-Wagner interfacial polarization. At lower frequencies, the charge carriers have sufficient time to migrate and accumulate at the interfaces, thereby contributing significantly to the dielectric loss. With increasing frequency, the charge carriers become increasingly unable to follow the rapidly alternating electric field, resulting in a reduction in tanδ. This behavior is characteristic of heterogeneous dielectric systems containing grains and grain boundaries. The decrease in dielectric loss with increasing frequency is also linked with the hopping of charge carriers between Fe^{2+} and Fe^{3+} ions at the octahedral sites of the inverse spinel Fe_3O_4 lattice. At higher frequencies, only the faster polarization mechanisms can respond effectively to the applied field, while the slower space-charge contribution becomes progressively suppressed. Therefore, the dielectric loss approaches lower values in the higher-frequency region.

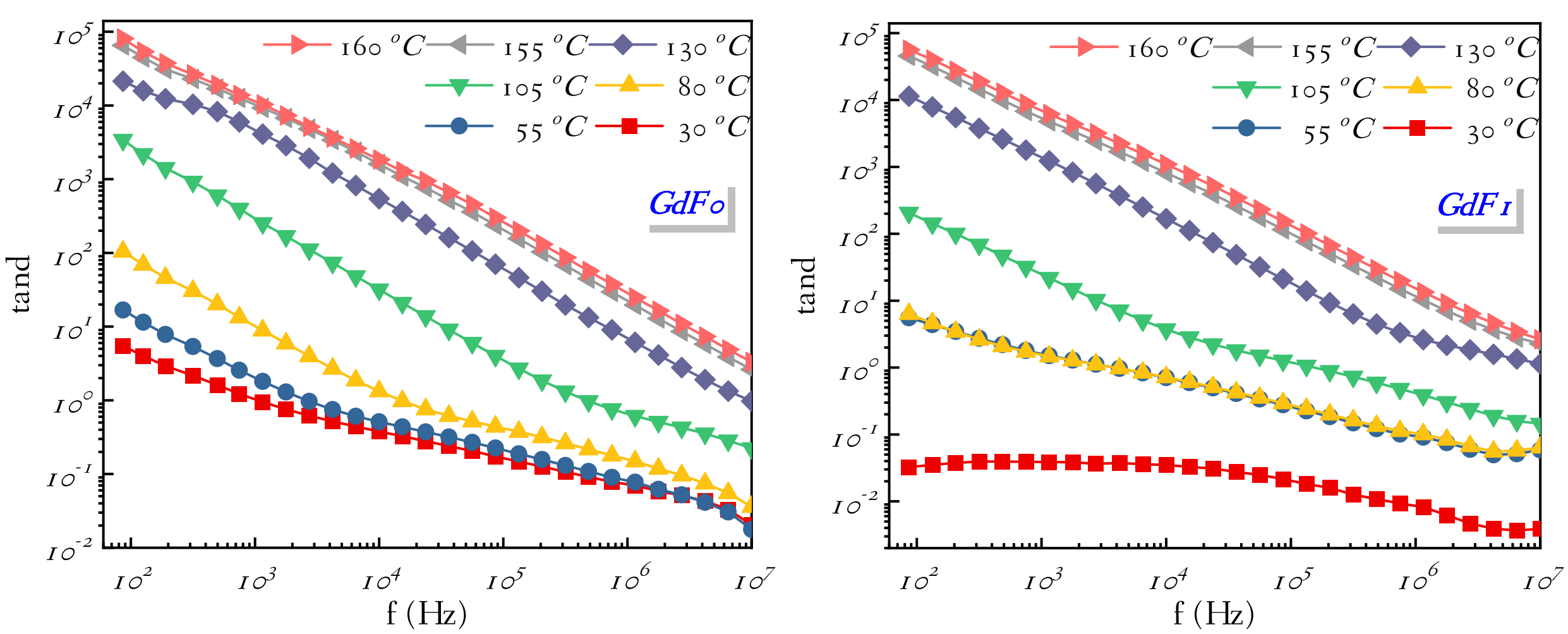

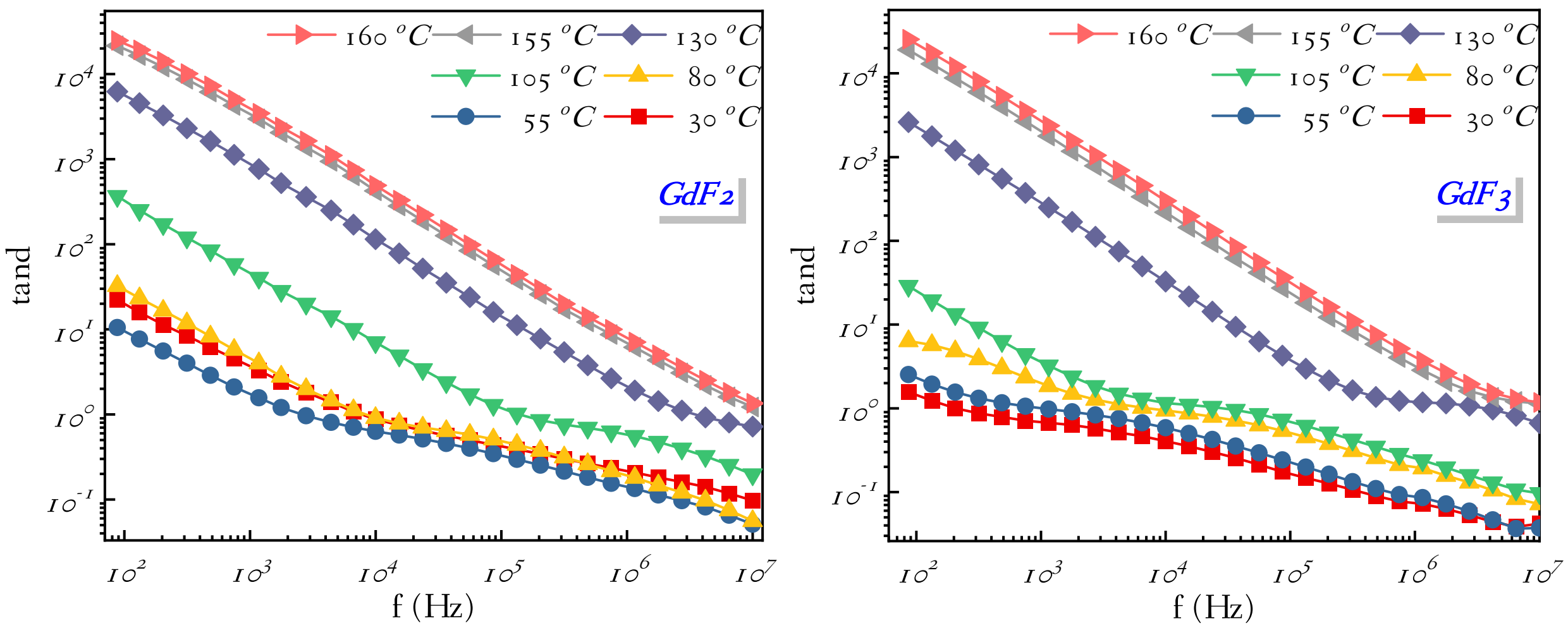

Figure S2: *Variation of a dielectric loss in all samples.*

A systematic decrease in tanδ is observed with Gd incorporation for the investigated compositions, although the magnitude of the change depends on frequency and temperature. The incorporation of Gd^{3+} modifies the local lattice environment and defect structure of Fe_3O_4, thereby influencing charge-carrier hopping and interfacial polarization. The reduced tanδ at higher Gd concentrations can therefore be associated with the modified charge transport and polarization processes introduced by Gd substitution. The temperature dependence further supports a thermally activated conduction mechanism. As temperature increases, charge carriers acquire sufficient thermal energy to overcome potential barriers at grain boundaries and participate more readily in hopping and interfacial polarization processes. This results in enhanced dielectric loss, particularly in the lower-frequency region. The broad frequency dispersion and absence of a sharp relaxation peak indicate a non-Debye-type dielectric response, consistent with the heterogeneous nature of the Fe_3O_4 nanocrystals.

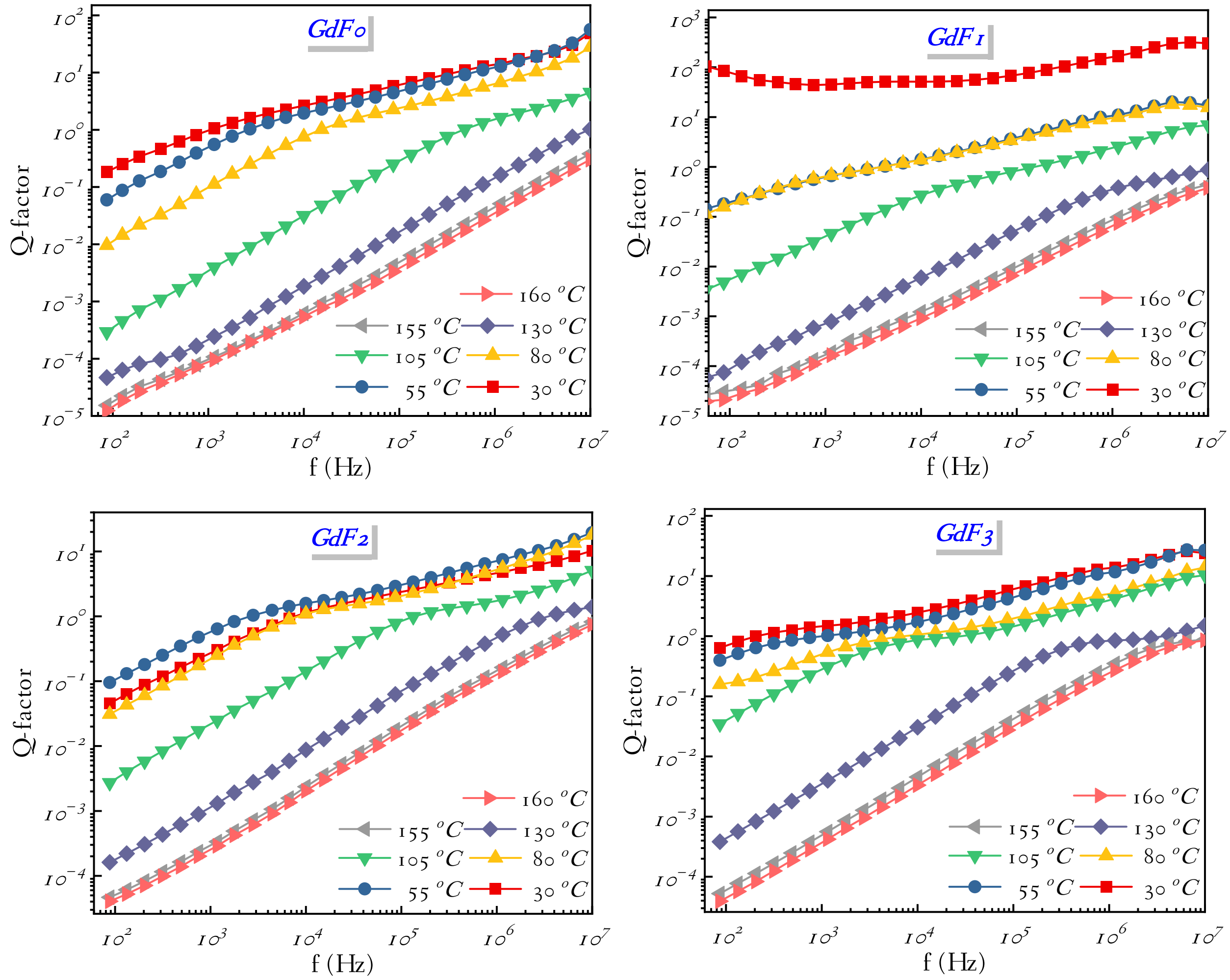

Figure S3: *Frequency-dependent variation of the quality factor of Fe_3O_4 nanocrystals at different temperatures.*

The quality factor (Q) was further evaluated from the dielectric loss, using Q = 1/tan δ, with Gd doping at different temperatures, as shown in Figure S3. Since Q is inversely related to tanδ, the variation in Q provides complementary information on the dielectric loss and energy-dissipation characteristics of the samples. For most compositions, the Q-factor increases with frequency, particularly at higher frequencies, reflecting reduced dielectric loss. This behavior indicates reduced dielectric energy dissipation as the contribution of slow space-charge/interfacial polarization becomes less pronounced at higher frequencies. The magnitude and frequency dependence of Q also vary with Gd concentration

and temperature, demonstrating that Gd incorporation modifies the polarization and charge-transport processes in the Fe_3O_4 nanocrystals.

2.3. Electrical Activation Energy

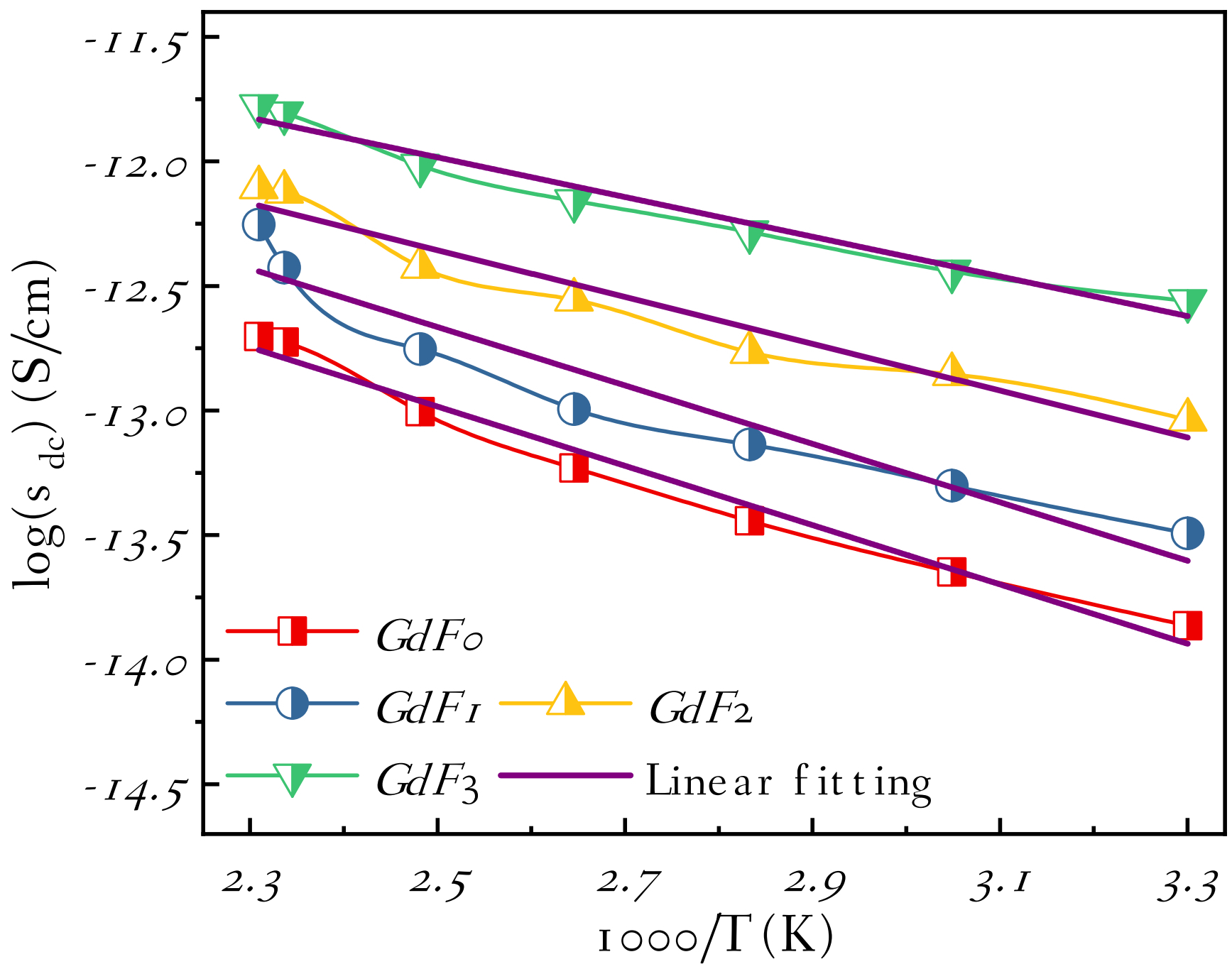

Figure S4: *DC conductivity versus 1000/T and activation energy.*
